# The RSK2-RPS6 axis promotes axonal regeneration in the peripheral and central nervous systems

**DOI:** 10.1371/journal.pbio.3002044

**Published:** 2023-04-17

**Authors:** Charlotte Decourt, Julia Schaeffer, Beatrice Blot, Antoine Paccard, Blandine Excoffier, Mario Pende, Homaira Nawabi, Stephane Belin

**Affiliations:** 1 Univ. Grenoble Alpes, Inserm, U1216, Grenoble Institut Neurosciences, 38000 Grenoble, France; 2 Institut Necker Enfants Malades, INSERM U1151, Université de Paris, Paris, France; University of Notre Dame, Center for Stem Cells and Regenerative Medicine, UNITED STATES

## Abstract

Unlike immature neurons and the ones from the peripheral nervous system (PNS), mature neurons from the central nervous system (CNS) cannot regenerate after injury. In the past 15 years, tremendous progress has been made to identify molecules and pathways necessary for neuroprotection and/or axon regeneration after CNS injury. In most regenerative models, phosphorylated ribosomal protein S6 (p-RPS6) is up-regulated in neurons, which is often associated with an activation of the mTOR (mammalian target of rapamycin) pathway. However, the exact contribution of posttranslational modifications of this ribosomal protein in CNS regeneration remains elusive. In this study, we demonstrate that RPS6 phosphorylation is essential for PNS and CNS regeneration in mice. We show that this phosphorylation is induced during the preconditioning effect in dorsal root ganglion (DRG) neurons and that it is controlled by the p90S6 kinase RSK2. Our results reveal that RSK2 controls the preconditioning effect and that the RSK2-RPS6 axis is key for this process, as well as for PNS regeneration. Finally, we demonstrate that RSK2 promotes CNS regeneration in the dorsal column, spinal cord synaptic plasticity, and target innervation leading to functional recovery. Our data establish the critical role of RPS6 phosphorylation controlled by RSK2 in CNS regeneration and give new insights into the mechanisms related to axon growth and circuit formation after traumatic lesion.

## Abstract 2

This study shows that RSK2-controlled phosphorylation of RPS6 on Ser233/236 is critical for peripheral and central nervous system regeneration and functional recovery through modulation of spinal cord plasticity and target innervation.

## Introduction

In contrast to developing neurons or the ones from the peripheral nervous system (PNS), mature neurons from the central nervous system (CNS) fail to regenerate their axons after an insult (neurodegenerative diseases or traumatic lesions). Patients must bear irreversible and permanent motor, cognitive and/or sensory disabilities. The continuous increase of such nervous system disorders worldwide, along with the lack of efficient therapies, makes axon regeneration and functional recovery major challenges of public health.

CNS regenerative failure has both extrinsic and neuronal intrinsic components [1,2]. The mTOR (mammalian target of rapamycin) pathway is one of the key neuronal signaling pathway controlling axon regeneration. Indeed, it has been shown that the activation of mTOR pathway via PTEN (**P**hosphatase and **TEN**sin homolog) deletion in neurons, triggers robust axon regeneration in the visual system and in the corticospinal tract [3–7]. Subsequently, combinatorial/synergistic approaches, which often include mTOR pathway activation, have led to long-distance regeneration [8,9]. Additionally, the analysis of specific retinal ganglion cells (RGC) subpopulations regenerative capacity revealed osteopontin and IGF as regulators of axon regeneration through mTOR activation [10]. In the PNS, mTOR has also been shown to regulate axon regeneration. However, its exact contribution to this process remains unclear. Indeed, one target of mTOR, S6 kinase 1 (S6K1), inhibits axon regeneration through negative feedback on mTOR [11,12]. In contrast, TSC2 genetic deletion or PTEN inhibition (negative regulators of mTOR pathway) leads to a modest increase of axon regeneration after sciatic nerve lesion [13–15]. Moreover, pharmacological inhibition of mTOR, in cultured DRG (dorsal root ganglia) neurons induces only a mild effect [16,17].

One major readout of mTOR activation is the phosphorylation of the ribosomal protein S6 (RPS6) [18], which belongs to the small 40S subunit of the ribosome, the functional unit of protein synthesis. RPS6 is an RNA-binding protein that stabilizes the ribosome by interacting with the ribosomal RNA [19]. Among all ribosomal proteins, RPS6 has attracted most attention as it was the first one shown to have inducible posttranslational modifications [20]. For almost 40 years, RPS6 phosphorylation has been studied, yet, many unknowns remain about its physiological functions [21]. Interestingly, in the retina, RGC subpopulations that are the most resilient to injury have high endogenous levels of RPS6 phosphorylation, which is maintained after injury [10]. Nevertheless, whether this phosphorylation is directly associated with mTOR activation remains elusive. Moreover, in some cases, injury signals may trigger specific events to prime neurons towards a pro-regenerative response. This feature has been elegantly described in the model of the dorsal column lesion in the spinal cord [22]. As part of the CNS, the dorsal column, formed by the central branch of DRG neurons, is not able to regenerate after spinal cord injury. However, a prior lesion of the DRG peripheral branch, which forms, for example, the sciatic nerve at the lumbar level, primes DRG neurons to regenerate their axon in the central branch: this is called the preconditioning effect [23,24]. Interestingly, the level of RPS6 phosphorylation increases in DRG neurons after sciatic nerve injury [10,18].

Altogether, the phosphorylation status of RPS6 stands as critical to promote axon regeneration. Yet, the exact role of RPS6 phosphorylation and the mechanisms regulating this posttranslational modification in the process of CNS regeneration remain elusive. Surprisingly, mTOR inhibition in DRG cultures does not impact RPS6 phosphorylation [17]. This result suggests that other signaling pathways might be controlling RPS6 phosphorylation, beside the mTOR pathway.

Proteins from the p90 S6 kinase (RSK) family are also known regulators of RPS6 phosphorylation [25]. The RSK protein family is composed of 4 isoforms (RSK1-4), with high homology (from 80% to 87%) [26]. RSK are mostly activated by extracellular signal-regulated kinase (ERK) and regulate important processes in cells, such as growth, survival, proliferation, and cell cycle progression [26]. Interestingly, Mao and colleagues found that RSK1 contributes to axon regeneration through activation of pro-regenerative proteins [27]. However, the contribution of the RSK-RPS6 axis in CNS regeneration has not been addressed yet.

In this study, we focus on the mouse lumbar DRG as a model of central and peripheral nervous system regeneration. We analyze a mouse line with unphosphorylable RPS6 to decipher its impact on regeneration. We show that RPS6 phosphorylation on Ser 235–236 residues is essential not only for PNS regeneration but also for the preconditioning effect. Among the 4 RSK, RSK2 is strongly expressed by DRG and its expression is regulated by axon injury. We further show that RSK2 modulates RPS6 phosphorylation to promote spinal cord regeneration, spinal synaptic plasticity, target innervation, and functional recovery in mice. Together, our results provide evidence that the RSK2/RPS6 axis is critical in nervous system regeneration.

## Results

### RPS6 phosphorylation on Ser 235–236 controls the preconditioning effect and contributes to sciatic nerve regeneration

**Fig 1:**
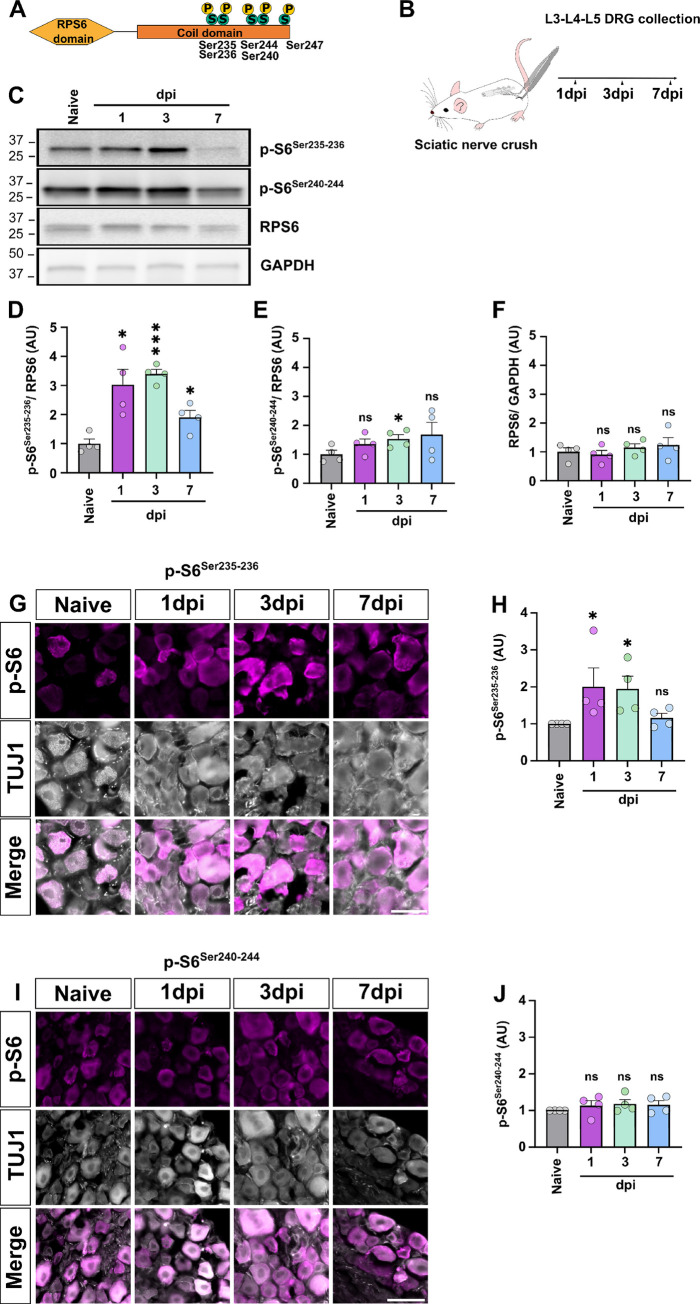
Specific RPS6 phosphorylation on Ser235-236 is induced by sciatic nerve injury. (A) Schematic representation of the 5 Serine (Ser) phosphorylation sites on the RPS6 coil domain. (B) Schematic representing the experimental workflow. (C) Representative western blot showing increase of RPS6 phosphorylation on Ser235-236 at 1 and 3 dpi while total RPS6 and GAPDH expressions remain stable. (D, E) Graphs showing the quantification of RPS6 phosphorylation on Ser235-S236 (D) on Ser240-244 (E) normalized to total RPS6. (F) Graph showing the quantification of total RPS6 normalized on GAPDH from C (mean ± SEM, Welch ANOVA test multiple comparisons, *N* = 4 animals per group). (G) Representative microphotographs of DRG sections stained with anti-p-S6^Ser235-236^ (in magenta) and anti-Tuj1 (in gray) in intact and at different time points upon sciatic nerve injury. Scale bar: 50 μm. (H) Graphs showing the quantification of G confirming western blot data with an increase of p-S6^Ser235-236^ at 1 and 3 dpi (mean ± SEM, Krukal–Wallis test multiple comparisons, *N* = 4 animals per group). (I) Representative microphotographs of DRG sections labeled with anti p-S6^Ser240-244^ (in magenta) and anti-Tuj1 (in gray) in intact and at different time points upon sciatic nerve injury. Scale bar: 50 μm. (J) Graphs showing the quantification of I (mean ± SEM, Krukal–Wallis test multiple comparisons, *N* = 4 animals per group). ⁎⁎⁎*p* < 0.001, ⁎⁎*p* < 0.01, ⁎*p* < 0.05, ns: not significant. Raw data can be found in Supporting information (S1 Data and S1 Raw Images). dpi, days post-injury; DRG, dorsal root ganglion; RPS6, ribosomal protein S6.

The phosphorylation of ribosomal protein RPS6 is often used as a readout of mTOR activation [18]. However, the exact contribution of RPS6 during regeneration has never been addressed. Indeed, RPS6 is a core ribosomal protein that carries 5 serine residues that can be phosphorylated (Ser235, Ser236, Ser240, Ser244, and Ser 247) [19,21] (**Fig 1A**). To understand the role of RPS6 during axon regeneration, we analyzed its dynamics of phosphorylation upon sciatic nerve injury (**Fig 1B**). We collected 6-week-old wild-type mice lumbar dorsal root ganglia (DRG-L3 to L5) from intact (naive) condition and 1, 3, and 7 days post-sciatic nerve injury (dpi). Western blot analysis using specific anti-p-S6^Ser235-236^ and anti-p-S6^Ser240-244^ antibodies revealed that RPS6 phosphorylation on Ser235-236 is up-regulated at 1 dpi and reaches a peak at 3 dpi, before decreasing at 7 dpi (**Fig 1C and 1D**). On the other hand, RPS6 phosphorylation on Ser240-244 remains overall stable, despite a slight increase only at 3 dpi (**Fig 1E**). The total level of RPS6 remains stable after injury (**Fig 1F**). In parallel, we analyzed the levels of phosphorylated RPS6 in DRG sections using immunofluorescence. Consistently with the western blot analysis, we observed an increase of p-S6^Ser235-236^ expression from 1 dpi, with a peak at 3 dpi. At 7 dpi, the level of p-S6^Ser235-236^ was back to the control (uninjured) condition (**Fig 1G and 1H**). Conversely, the level of p-S6^Ser240-244^ did not display any significant change over time (**Fig 1I and 1J**). Together, these results show that RSP6 phosphorylation on Ser235-236 is up-regulated in DRG upon sciatic nerve injury.

Then, we asked whether RPS6 phosphorylation is differentially regulated in DRG neuronal subpopulations. We analyzed p-S6^Ser235-236^ expression intensity across different subpopulations of DRG (**S1A Fig**). In intact condition, we found that all the analyzed neuronal subpopulations have basal levels of p-S6^Ser235-236^. This is in sharp contrast with the CNS, where only few subpopulations maintain p-S6^Ser235-236^ expression in mature neurons (for example, the alpha RGC or the intrinsic photosensitive RGC in the retina [10]). Interestingly, 3 days upon sciatic nerve injury, p-S6^Ser235-236^ is differentially regulated in these subpopulations. Most of these populations up-regulate p-S6^Ser235-236^ (**S1B and S1C Fig**): Islet1/2^+^ and Advillin^+^ (most of sensory neurons), TrkB^+^ (mechanoreceptor neurons), and Parvalbumin^+^ (proprioceptive neurons) DRG neurons. For some few others, p-S6^Ser235-236^ is not statistically modulated by sciatic nerve injury: TrkA^+^ (nociceptive neurons), Calbindin^+^ (mechanosensitive neurons), and Somatostatin^+^ (itch-sensing neurons) neurons. However, we noticed a trend for p-S6^Ser235-236^ increased phosphorylation for TrkA^+^ and Somatostatin^+^ DRG populations (**S1B and S1C Fig**).

In order to assess the regenerative abilities of these subpopulations, we retro-labeled regenerating DRG neurons with intranervous injection of Alexa-555 conjugated cholera toxin B (CTB) after sciatic nerve crush. All subpopulations of neurons regenerate, as we found CTB^+^ neurons across all the analyzed subpopulations. Regenerative DRG subpopulations ratio corresponds to the naive condition, except for TrkA^+^ and TrkB^+^ that show reversed ratios (**S1D Fig**). These results are also in contrast to what has been reported in the CNS, where only few subpopulations display survival and regeneration capabilities [10].

Then, we addressed the contribution of RPS6 phosphorylation to axon regeneration. We analyzed the regenerative response of a transgenic mouse line that endogenously carries an unphosphorylable version of RPS6 [28]. In this mouse line, all Serine phosphorylation sites (Ser235, 236, 240, 244, and 247) are mutated to Alanine (**S2A Fig).** We verified that RPS6 cannot be phosphorylated using immunostaining on DRG sections (**S2B Fig**). We then extracted proteins from intact and 3 dpi DRG from 6-week-old wild-type (RPS6^p+/p+^), heterozygous (RPS6^p+/p-^), and homozygous (RPS6^p-/p-^) littermates. Western blot analysis using anti-p-S6^Ser235-236^ and anti-p-S6^Ser240-244^ antibodies validated that RPS6 is not phosphorylated, neither on Ser235-236 nor in Ser240-244 sites, in intact and 3 dpi DRG from homozygous mutant mice (RPS6^p-/p-^), in contrast to RSP6^p+/p+^ and RPS6^p+/p-^ littermates (**S2C–S2E Fig**). The total level of RPS6 was used as a control and did not differ between all genotypes. Consistently with the peaked expression of p-S6^Ser235-236^ at 3 dpi (**Fig 1C and 1D**), we observed a significant increase of p-S6^Ser235-236^ both in RPS6^p+/p+^ and in RPS6^p+/p-^ mice at 3 dpi (**S2D Fig**). On the other hand, no change was observed in the level of p-S6^Ser240-244^ phosphorylation at 3 dpi (**S2E Fig**).

**Fig 2:**
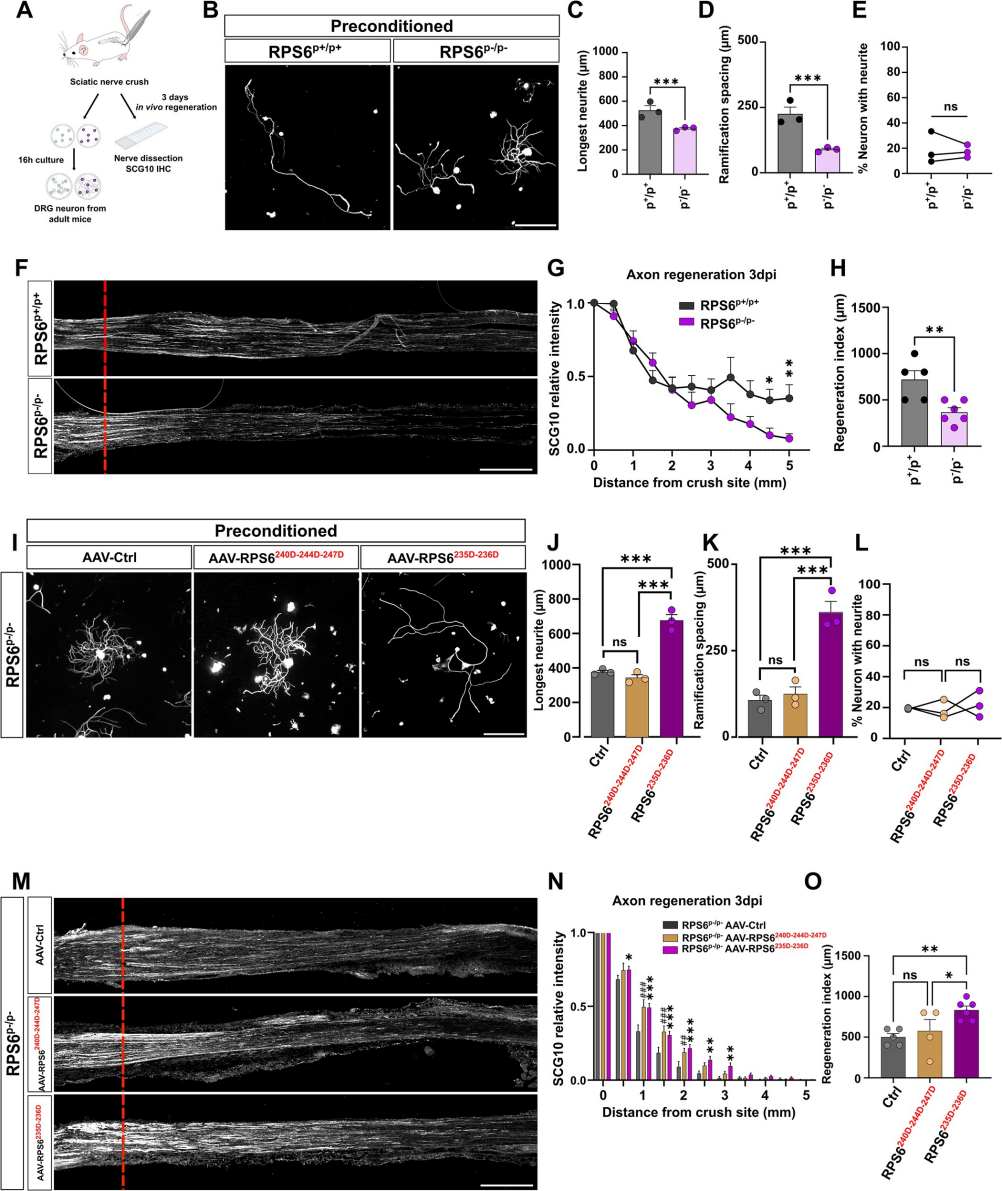
RPS6 phosphorylation on Ser 235–236 is key for axon regeneration. (A) Workflow of unphosphorylable RPS6 mouse line analysis in the paradigm of preconditioning and sciatic nerve regeneration. (B) Representative microphotographs of preconditioned cultures of mature DRG neurons from WT (RPS6^p+/p+^) and homozygous (RPS6^p-/p-^) mice defective for RPS6 phosphorylation showing that the preconditioning effect is inhibited in RPS6^p-/p^ mice. Scale bar: 250 μm. (C–E) Graphs showing the quantification of B. (C) Longest neurite length per neuron, 16 h after plating (mean ± SEM, unpaired *t* test, 3 independent DRG cultures, approximately 50 cells counted per condition per culture). (D) Distance between 2 ramifications of longest neurite (mean ± SEM, unpaired *t* test, 3 independent DRG cultures, approximately 50 cells counted per condition per culture). (E) Percentage of neurons growing a neurite 16 h after plating (mean ± SEM, unpaired *t* test, 10 random microscopy fields quantified per condition per culture). (F) Representative confocal images of the sciatic nerve sections 3 days post-injury from WT (RPS6^p+/p+^) and homozygous (RPS6^p-/p-^) mice. Regenerating axons are labeled with anti-SCG10 antibody (white). The red dashed line indicates the injury site. Scale bar: 500 mm. (G) Quantification of regenerative axons 3 dpi from F (mean ± SEM, unpaired multiple *t* test, *N* = 7–8 animals per group); (H) 3 dpi regeneration index of F (mean ± SEM, unpaired *t* test, at least 5 animals per condition). (I) Representative microphotographs of preconditioned cultures of mature DRG neurons from RPS6^p-/p-^ mice overexpressing RPS6^Ser235D-236D^ or RPS6^Ser240D-244D-247D^. Only RPS6^Ser235D-236D^ overexpression restores the preconditioning effect phenotype. Scale bar: 250 μm. (J–L) Graphs showing quantification of I. (J) Longest neurite length per neuron, 16 h after plating (mean ± SEM, Ordinary one-way ANOVA, 3 independent DRG cultures, approximately 50 cells counted per condition per culture). (K) Distance between 2 ramifications of longest neurite (mean ± SEM, Ordinary one-way ANOVA, 3 independent DRG cultures, approximately 50 cells counted per condition per culture). (L) Percentage of neurons growing a neurite 16 h after plating (mean ± SEM, two-way ANOVA, 10 random microscopy fields quantified per condition per culture). (M) Representative confocal images of RPS6^p-/p-^ sciatic nerve sections 3 days post-injury after AAV8-Ctrl, AAV8-RPS6^Ser235D-236D^, or AAV8-RPS6^Ser240D-244D-247D^ overexpression. Regenerating axons are labeled with anti-SCG10 antibody (white). The red dashed line indicates the injury site. Scale bar: 500 mm. (N) Quantification of regenerative axons 3 dpi from M (mean ± SEM, two-way ANOVA multiple comparisons, *N* = at least 3 animals per group) (⁎, ⁎⁎, ⁎⁎⁎: comparison between groups AAV8-Ctrl and AAV8-RPS6^Ser235D-236D^; #, ##; ###: comparison between groups AAV8-Ctrl and AAV8-RPS6^Ser240D-244D-247D^), and (O) 3 dpi regeneration index (mean ± SEM, Ordinary one-way ANOVA, at least 5 animals per condition). ⁎⁎⁎*p* < 0.001, ⁎⁎*p* < 0.01, ⁎*p* < 0.05. ###*p* < 0.001, ##*p* < 0.01, #*p* < 0.05. ns: not significant. Raw data can be found in Supporting information (S1 Data). dpi, days post-injury; DRG, dorsal root ganglion; RPS6, ribosomal protein S6.

Next, we asked whether RPS6 phosphorylation was required for the preconditioning effect. To do so, we performed sciatic nerve injury unilaterally on RPS6^p+/p+^ and RPS6^p-/p-^ mice. Three days later, we isolated L3 to L5 DRG neurons from the intact (naive) side and injured (preconditioned) side, and cultured them for 16 h (**Fig 2A**). In naive condition, neurons from RPS6^p+/p+^ and RPS6^p-/p-^ mice have short, highly ramified neurites (**S2F Fig**). Indeed, we found no significant difference in longest neurite length nor in ramification spacing between RPS6^p+/p+^ and RPS6^p-/p-^ naive DRG neurons (**S2F–S2I Fig**). As expected, in the preconditioned cultures (after sciatic nerve injury), DRG neurons from RPS6^p+/p+^ mice have the typical phenotype of preconditioned neurons, with long neurites and few ramifications (**Fig 2B**). Strikingly, in RPS6^p-/p-^ preconditioned DRG neurons, neurites are short and highly ramified. They present the same phenotype as naive DRG neurons without prior sciatic nerve injury (**Fig 2B–2E**). In summary, this experiment shows that RPS6 phosphorylation is required for the preconditioning effect.

Then, we asked whether RPS6 phosphorylation was involved in PNS regeneration. To this end, we performed sciatic nerve injury on 6-week-old RPS6^p+/p+^ and RPS6^p-/p-^ mice and analyzed the extent of regeneration at 3 dpi, by SCG10 immunostaining on sciatic nerve sections. Interestingly, while RPS6^p+/p+^ and RPS6^p-/p-^ mice have the same number of axons regenerating in the sciatic nerves, we found that axons from RPS6^p-/p-^ grew significantly less (**Fig 2F–2H**). This result suggests that RPS6 phosphorylation is involved in long-distance growth of regenerating PNS axons.

To better assess the contribution of p-S6^Ser235-236^ and p-S6^Ser240-244-247^ in the preconditioning effect and sciatic nerve regeneration, we generated specific phosphomimic constructs. In the RPS6^Ser235D-236D^ construct, Ser240, Ser244, and Ser247 have been replaced by an Alanine (not phosphorylable) and Ser235-Ser236 by an Aspartic acid (D) to mimic a constitutive phosphorylation. For the RPS6^Ser240D-244D-247D^, Ser235 and Ser236 have been replaced by an Alanine (not phosphorylable) and Ser240, Ser244, and Ser247 by an Aspartic acid (D) to mimic a constitutive phosphorylation. We verified their incorporation in ribosomes by performing cytoplasmic ribosome purification from N2A cells transfected with these plasmids (**S3A and S3B Fig**). Both constructs are expressed and incorporated into ribosomes.

We generated AAV8 viruses from these constructs and injected them intrathecally in WT mice (**S3C–S3F Fig**). We found that RPS6^Ser240D-244D-2447D^ has no effect on the morphology of naive DRG neurons. In contrast, overexpression of RPS6^Ser235D-236D^ mimics the preconditioning effect with neurons presenting long neurites and few ramifications (**S3C–S3F Fig**). In parallel, we tested their effects on sciatic nerve regeneration on WT mice. While p-S6^Ser240D-244D-2447D^ exhibits only a mild effect, RPS6^Ser235D-236D^ significantly enhances axon regeneration at long distances (**S3G and S3H Fig**). However, for the regeneration index (RI50), we do not see any statistically significant difference (**S3I Fig**).

We performed the same experiments on RPS6^p-/p-^ mice (**Figs 2I–2O and S3J–S3M**). RPS6^Ser240D-244D-247D^ is not able to rescue the RPS6^p-/p-^ phenotype. In contrast, RPS6^Ser235D-236D^ rescues the RPS6^p-/p-^ phenotype by restoring the preconditioning effect phenotype (**Fig 2I–2L**). Moreover, overexpression of RPS6^Ser235D-236D^ enhances sciatic nerve regeneration RPS6^p-/p-^ mice (**Fig 2M–2O**).

Altogether, our results show that RPS6 phosphorylation on Ser235-236 is a major effector of the preconditioning effect and axon regeneration. The Ser240-244 phosphorylation might play a role in the physiology of these neurons and contributes modestly to axon regeneration process.

### RPS6 phosphorylation and preconditioning effect are not controlled by the mTOR pathway

**Fig 3:**
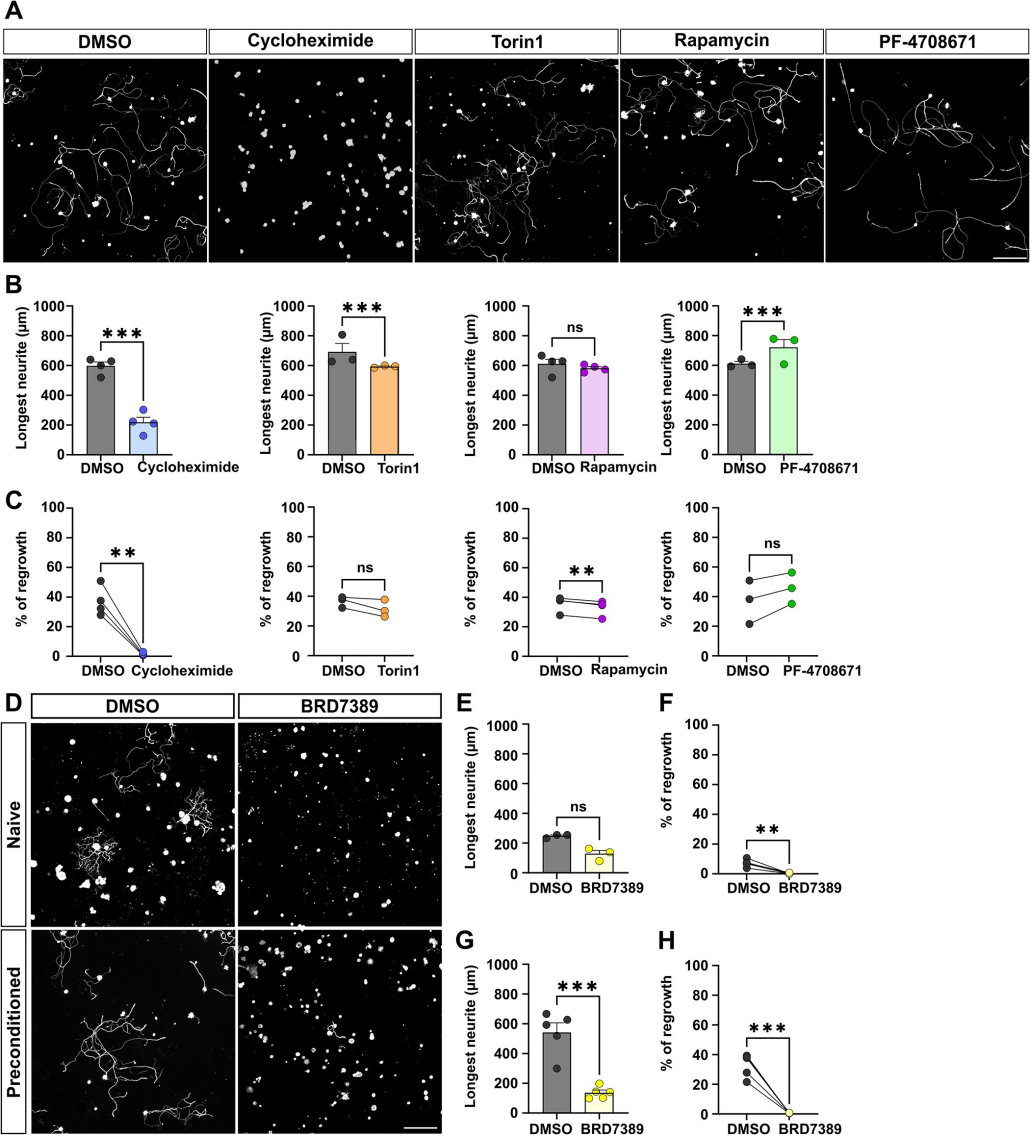
RSK controls the preconditioning effect in mature DRG neurons. (A) Representative microphotographs of preconditioned cultures of mature DRG neurons treated with DMSO (control), translation inhibitor (Cycloheximide, 2 nM), mTOR inhibitors (Torin1, 5 nM or Rapamycin, 0.1 nM), S6K1 inhibitor (PF-4708671-8uM). Scale bar: 250 μm. (B) Quantification of longest neurite length per neuron 16 h after plating (mean ± SEM, two-way ANOVA, 3–4 independent DRG cultures, approximately 50–100 cells counted per condition per culture (except for Cycloheximide)). (C) Percentage of neurons growing a neurite 16 h after plating (mean ± SEM, two-way ANOVA, 10 random microscopy fields quantified per condition). (D) Representative microphotographs of naive and preconditioned cultures of mature DRG neurons treated with DMSO (control) or RSK inhibitor (BRD-7389 (3 μm)). Scale bar: 250 μm. (E) Quantification of longest neurite length per neuron 16 h after plating in naive condition (mean ± SEM, two-way ANOVA, 3 independent DRG cultures, approximately 50–100 cells counted per condition per culture for DMSO condition; all neurons found with a neurite were quantified in BRD7389 condition). (F) Percentage of neurons growing a neurite 16 h after plating in naive DRG treated with DMSO or BRD7389 (mean ± SEM, paired *t* test, 10 random microscopy fields quantified per condition). (G) Quantification of the longest neurite length 16 h after plating in PC DRG treated with DMSO or BRD7389 (mean ± SEM, two-way ANOVA, approximately 50–100 cells per condition per culture for DMSO condition; all neurons growing a neurite were quantified in BRD7389 condition). (H). Percentage of neurons growing a neurite 16 h after plating in naive DRG treated with DMSO or BRD7389 (mean ± SEM, paired *t* test, 5 independent DRG cultures, 10 random microscopy fields were quantified per condition). ⁎⁎⁎*p* < 0.001, ⁎⁎*p* < 0.01, ⁎*p* < 0.05. Raw data can be found in Supporting information (S1 Data). DRG, dorsal root ganglion; mTOR, mammalian target of rapamycin; PC, precondtionned.

RPS6 phosphorylation is commonly used as a readout of mTOR pathway activation, particularly during nervous system regeneration [3,8]. As RPS6 phosphorylation is key for the preconditioning effect and sciatic nerve regeneration, we asked which signaling pathway controls its phosphorylation in DRG. To do so, we used a pharmacological approach. We performed sciatic nerve crush unilaterally on wild-type mice and 3 days later, we isolated DRG neurons to put them in culture. We used Cycloheximide as a global inhibitor of translation, Rapamycin and Torin1 as inhibitors of mTOR, PF-4708671 as a S6 kinase inhibitor, BRD7389 as an inhibitor of the p90 RSK (**S4A Fig**), and DMSO as control [29]. One hour after plating, we treated cultures with the drug of interest, then we assessed neurite growth after 16 h. We found that Cycloheximide-mediated inhibition of global translation totally blocks neurite outgrowth, both in naive and in preconditioned DRG cultures (**Figs 3A–3C and S4B–S4D**). This result shows that protein translation is key for neurite outgrowth in naive and preconditioned cultures.

Interestingly, in naive conditions, inhibition of mTOR or S1 kinase did not prevent neurite outgrowth (**S4B–S4D Fig**). We found no difference in the length of the longest neurite nor in the total number of neurons that grow a neurite between control and mTOR inhibition (Torin1, Rapamycin) treatments (**S4C and S4D Fig**). Inhibition of S6K with PF-4708671 caused a slight increase of the number of neurons that grow a neurite (6.7% ± 1.6% for DMSO versus 9.0% ± 1.2% for PF-4708671) (**S4C and S4D Fig**). In preconditioned DRG cultures, mTOR inhibition via Torin1 has a mild effect on the extent of growth (691 ± 58 μm for DMSO versus 594 ± 4 μm for Torin1) (**Fig 3A–3C**). Unlike Torin1, Rapamycin-treated DRG have fewer growing neurites (35.6% ± 2.6% for DMSO versus 25.8% ± 1.9% for Rapamycin) (**Fig 3A–3C**). When preconditioned neurons are treated with S6K inhibitor, a slight increase of the longest neurite length is observed (610 μm ± 15 for DMSO versus 694 μm ± 13 for PF-4708671) but the total number of neurons growing a neurite is unchanged (**Fig 3A–3C**). Altogether, our results show that mTOR nor its downstream effector S6K1 are the main actors of the preconditioning effect.

Strikingly, the inhibition of the RSK family with BRD7389 completely blocked neurite outgrowth, both in naive DRG cultures and in preconditioned DRG cultures (**Fig 3D–3H**). We verified that this effect was not due to drug toxicity as the number of Tuj1-positive cells is similar between DMSO and BRD7389 treatments. This result suggests that RSK is a family of kinases involved in the preconditioning effect.

### RSK2 expression is regulated by sciatic nerve injury and controls RPS6 phosphorylation in DRG neurons

As BRD7389 treatment shows a striking effect on neuronal growth, we next assessed the expression of RSK family members in adult DRG. In mice, RSK family is composed of 4 isoforms with high homology, particularly in the 2 kinase domains (**S5A and S5B Fig**). Therefore, we designed specific RNA probes that target unique and specific regions of each isoform (RSK1 to 4) (**S5C Fig and S1 Table**). We verified the specificity of these mRNA probes on adult brain sections for RSK1 to 3, where we found the expected labeling in the hippocampus [30]. As RSK4 is weakly expressed in adult tissues, we performed in situ hybridization on sagittal sections from E12,5 embryo. We found that RSK4 is specifically expressed in the ribs primordia as described in [31] (**S5D Fig**). We performed in situ hybridization on cryosections of adult DRG from 6-week-old wild-type mice (**S5E Fig**). We found that RSK 2 and 3 are enriched in DRG in intact conditions, whereas RSK1 is lowly expressed and RSK4 is not expressed (**S5F Fig**).

Then, we investigated whether the expression of RSK1-4 is modulated by axon injury. To do so, we collected DRG at different time points after sciatic nerve crush (**S5E Fig**). We found that RSK4, even after injury, is not expressed in DRG (**S5F Fig**). RSK1 is slightly up-regulated in few neurons as reported by Mao and colleagues [27]. *RSK3* mRNA expression is not modulated by the injury (**S5F Fig**). In contrast, RSK2 is specifically up-regulated by sciatic nerve injury at 1 dpi and 3 dpi, before decreasing back to the control (intact) level at 7 dpi. Therefore, we focused the rest of the study on RSK2.

**Fig 4:**
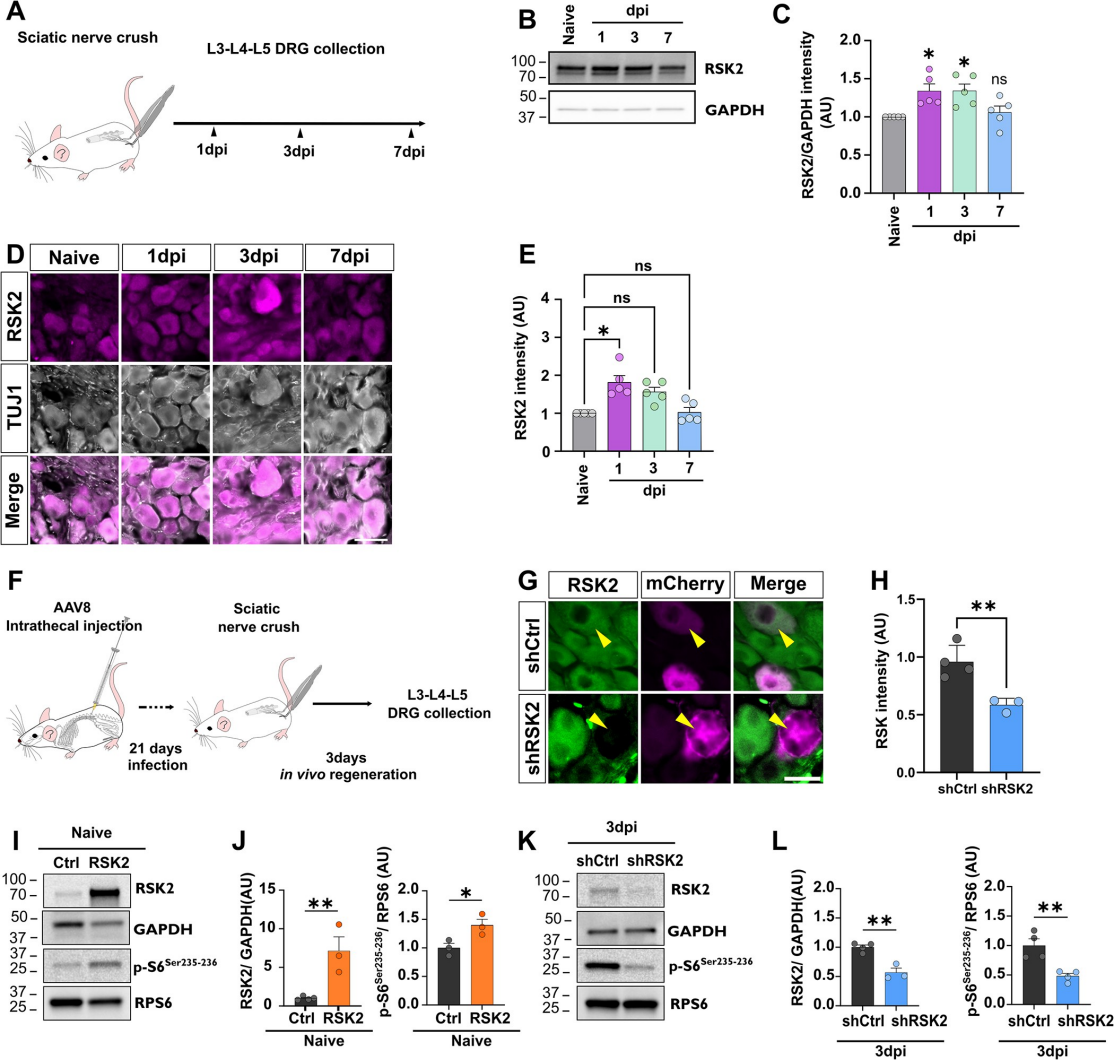
RSK2 regulates RPS6 Ser235-236 phosphorylation in mature DRG. (A) Workflow of experiment. (B) Western blot showing up-regulation of RSK2 expression upon sciatic nerve injury. (C) Quantification of B (mean ± SEM, one-sample *t* test, *N* = 5 animals per group). (D) Representative images of DRG sections from intact, 1 dpi, 3 dpi, or 7 dpi conditions labeled with anti-RSK2 (in magenta) and anti-Tuj1 (in gray). Scale bar: 50 μm. (E) Quantification of D (one-way ANOVA, Dunn’s multiple comparisons test, *N* = 5 animals per group). (F) Timeline of the experimental procedure to investigate in vivo the role of RSK2 in RPS6 phosphorylation. (G) Representative images of infected DRG by shCtrl or shRSK2 labeled with anti-RSK2 (in green) and anti-RFP (in magenta). Scale bar: 50 μm. (H) Quantification of G (mean ± SEM, unpaired *t* test, *N* = 3–4 animals per group). (I) Western blot showing that in vivo overexpression of RSK2, in naive DRG, induces RPS6 phosphorylation on Ser235-236 without sciatic nerve injury. (J) Quantification of I (mean ± SEM, one-sample *t* test, *N* = 3–4 animals per group). (K) Western blot showing in vivo inhibition of RSK2 in preconditioned DRG, 3 days after sciatic nerve injury, inhibits RPS6 phosphorylation on Ser235-236. (L) Quantification of K (mean ± SEM, one-sample *t* test, *N* = 3–4 animals per group). ⁎⁎⁎*p* < 0.001, ⁎⁎*p* < 0.01, ⁎*p* < 0.05, ns: not significant. Raw data can be found in Supporting information (S1 Data and S1 Raw Images). dpi, days post-injury; DRG, dorsal root ganglion; RPS6, ribosomal protein S6.

We then sought to determine the dynamics of RSK2 protein expression in DRG upon sciatic nerve injury (**Fig 4A**). By western blot (**Fig 4B and 4C**) and immunostaining (**Fig 4D and 4E**), using a specific anti-RSK2 antibody, we found a significant increase of RSK2 expression at 1 dpi and 3 dpi. At 7 dpi, its expression decreases back to control (intact) level. Importantly, RSK2 dynamics of expression (**Fig 4B–4E**) matches RPS6 dynamics of phosphorylation upon sciatic nerve injury (**Fig 1C–1D and 1G–1H**). This result supports the hypothesis that RSK2 is involved in RPS6 phosphorylation and in the control of the preconditioning effect.

In order to study the regulation of RPS6 phosphorylation by RSK2, we generated AAV viral vectors that either (i) overexpress RSK2; or (ii) knockdown specifically RSK2 expression with an shRNA-based silencing approach (shRSK2) (**S6A Fig**). We modulated RSK2 expression in DRG in vivo by intrathecal injection of AAV8-shRSK2 or AAV8-RSK2 in 4-week-old wild-type mice (**Figs 4F–4H and S6F and S6G**). In vivo overexpression of RSK2 in DRG significantly enhanced p-S6^Ser235-236^ in naive condition (**Fig 4I and 4J**), to the same level of RPS6 phosphorylation observed at 3 dpi (**Fig 1**).

For the silencing approach, we first verified the shRSK2 specificity by co-transfecting it with plasmids overexpressing RSK1, RSK2, RSK3, or RSK4 in N2A cells (**S6B–S6E Fig**). Western blot analysis confirmed that shRSK2 inhibits RSK2 expression only (**S6B–S6E Fig**). Three weeks after intrathecal injection of AAV8-shRSK2, 95% of DRG were infected with the virus (**S6F and S6G Fig**) and RSK2 expression was decreased by 50% (**Fig 4G and 4H**). Strikingly, RSK2 knockdown blocked the phosphorylation of RPS6 on Ser235-236 normally induced by sciatic nerve injury (**Fig 4K and 4L**). Together, our results highlight RSK2 as the main kinase that controls RPS6 phosphorylation on Ser235-236 in DRG upon sciatic nerve injury.

### RSK2 controls the preconditioning effect and sciatic nerve regeneration

**Fig 5:**
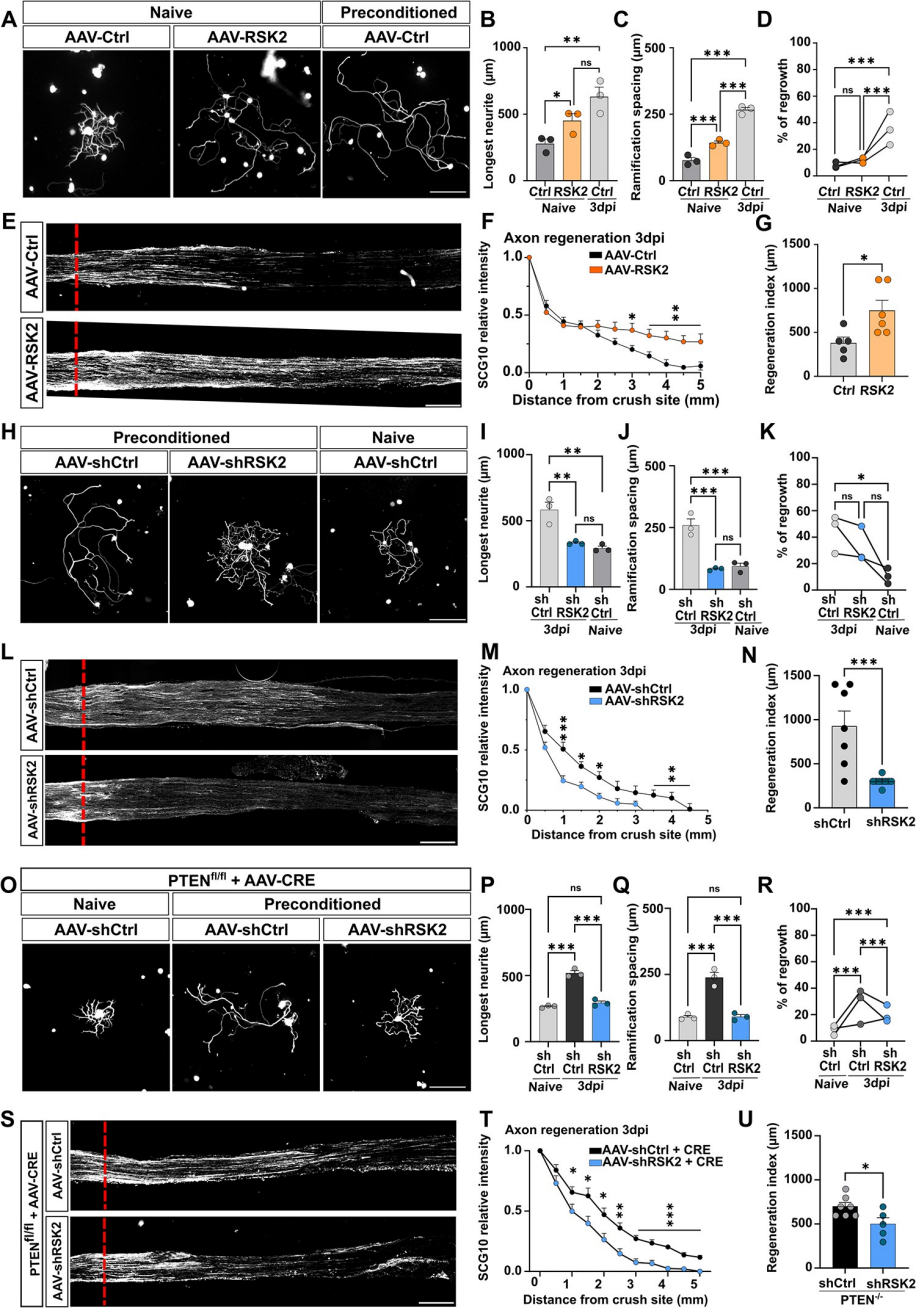
RSK2 controls the preconditioning effect and axon regeneration in the PNS. (A) Representative microphotographs of DRG dissociated cultures showing that RSK2 overexpression in naive cultures phenocopies the preconditioning effect. Scale bar: 250 μm. (B–D) Quantification of A. (B) Longest neurite length per neuron 16 h after plating (mean ± SEM, Ordinary one-way ANOVA, 3 independent DRG cultures, approximately 50–100 cells counted per condition per culture). (C) Mean distance between 2 ramifications (mean ± SEM, Ordinary one-way ANOVA, 3 independent DRG cultures, approximately 50 cells counted per condition per culture) and (D) percentage of neurons growing a neurite 16 h after plating (mean ± SEM, two-way ANOVA Tukey’s multiple comparisons test, 10 random microscopy fields quantified per condition per culture). (E) Representative images of the sciatic nerve sections 3 days post-injury from mice intrathecally injected with AAV8-PLAP (control) or AAV8-RSK2. Regenerating axons are labeled with anti-SCG10 antibody (white). The red dashed line indicates the injury site. Scale bar: 500 μm. (F) Quantification of regenerative axons from E (mean ± SEM, multiple *t* test, at least 6 animals per condition). (G) Regeneration index at 3 dpi (mean ± SEM, unpaired *t* test, at least 5 animals per condition). (H) Representative microphotographs of DRG dissociated cultures showing that RSK2 inhibition in preconditioned cultures phenocopies the naive condition. Scale bar: 250 μm. (I–K) Quantification of G. (I) Longest neurite length per neuron 16 h after plating (mean ± SEM, Ordinary one-way ANOVA, 3 independent DRG cultures, approximately 50–100 cells counted per condition per culture). (J) Mean distance between 2 ramifications (mean ± SEM, Ordinary one-way ANOVA, 3 independent DRG cultures, approximately 50 cells counted per condition per culture) and (K) percentage of neurons growing a neurite 16 h after plating (mean ± SEM, two-way ANOVA Tukey’s multiple comparisons test, 10 random microscopy fields were quantified per condition). (L) Representative images of the sciatic nerve sections 3 days post-injury from mice injected intrathecally with AAV-Sh-Scrambled or AAV-Sh-RSK2. Regenerating axons are labeled with anti-SCG10 antibody (white). The red dashed line indicates the injury site. (M) Quantification of regenerative axons from L (mean ± SEM, multiple *t* test, at least 6 animals per condition). (N) Regeneration index at 3 dpi (mean ± SEM, unpaired *t* test, at least 6 animals per condition). (O) Representative microphotographs of DRG dissociated cultures showing that RSK2 inhibition in PTEN deleted preconditioned cultures phenocopies the naive condition. Scale bar: 250 μm. (P–R) Quantification of O. (P) Longest neurite length per neuron 16 h after plating (mean ± SEM, Ordinary one-way ANOVA, 3 independent DRG cultures, approximately 50–100 cells counted per condition per culture). (Q) Mean distance between 2 ramifications (mean ± SEM, Ordinary one-way ANOVA, 3 independent DRG cultures, approximately 50 counted cells per condition per culture) and (R) percentage of neurons growing a neurite 16 h after plating (mean ± SEM, two-way ANOVA Tukey’s multiple comparisons test, 10 random microscopy fields quantified per condition). (S) Representative images of the sciatic nerve sections 3 days post-injury from mice injected intrathecally with AAV8-Sh-Scrambled or AAV8-Sh-RSK2 and AAV8-CRE in PTEN^fl/fl^ mice. Regenerating axons are labeled with anti-SCG10 antibody (white). The red dashed line indicates the injury site. (T) Quantification of regenerative axons from S (mean ± SEM, multiple *t* test, at least 6 animals per condition). (U) Regeneration index at 3 dpi (mean ± SEM, unpaired *t* test, at least 5 animals per condition). ⁎⁎⁎*p* < 0.001, ⁎⁎*p* < 0.01, ⁎*p* < 0.05, ns: not significant. Raw data can be found in Supporting information (S1 Data). DRG, dorsal root ganglion; PNS, peripheral nervous system.

Next, we asked whether RSK2 was involved in the preconditioning effect. To this end, we modulated RSK2 expression in vivo by intrathecal injection of AAV8 vectors and analyzed the neurite growth of both naive and preconditioned DRG in culture (**S7A Fig**). Strikingly, overexpression of RSK2 in vivo caused naive DRG to grow significantly longer neurites with fewer ramifications, a phenotype that is identical to the preconditioning effect (**Fig 5A–5D**). We found that this effect is specific of RSK2, as overexpression of RSK3 in naive DRG does not mimic the preconditioning effect (**S7B–S7E Fig**). Conversely, inhibition of RSK2 expression in vivo resulted in the loss of the preconditioning effect in DRG of the sciatic nerve injured side. Indeed, in absence of RSK2, preconditioned DRG neurons resemble the naive ones, with shorter, highly ramified neurites (**Fig 5H–5K**).

In parallel, we analyzed the regeneration of the sciatic nerve in vivo. We injected intrathecally AAV-RSK2, AAV-shRNA-RSK2, or corresponding controls in 4-week-old animals and performed unilateral sciatic nerve crush 3 weeks later **(S7A Fig)**. The extent of axon regeneration was analyzed by SCG10 immunostaining at 3 dpi. Similarly to the effect on DRG cultures, we found that RSK2 overexpression enhances sciatic nerve regeneration (**Fig 5E–5G**), with axons extending up to 5 mm from the lesion site. This effect is specific to RSK2, as overexpression of RSK3 did not affect sciatic nerve regeneration (**S7F–S7H Fig**). In contrast, RSK2 inhibition blocks axon regeneration in the sciatic nerve (**Fig 5L–5N**). Together, our results demonstrate that RSK2 is critical for peripheral nerve regeneration.

To show that mTOR and RSK2 act independently on the preconditioning effect, we activated the mTOR pathway through intrathecal injection of AAV-Cre in PTEN^f/f^ mice to delete PTEN [3]. We verified the efficiency of multiple AAV infections and the AAV8-Cre induced recombination by injecting a mix of AAV8-Cre and AAV-GFP intrathecally in the reporter mouse line STOP-tdTomato^f/f^ (**S7I and S7J Fig**). We found that almost 100% of neurons were expressing tdTomato, 2 weeks after injection and that 94% of these DRG were co-infected with AAV-Cre and AAV-GFP (**S7I and S7J Fig**). As expected, mTOR activation in naive DRG neurons does not induce the preconditioning effect (**Fig 5O–5R**). We then analyzed the axon growth outcome of RSK2 inhibition together with mTOR activation in preconditioned DRG neurons. We observed that mTOR activation does not modify the preconditioned effect. In contrast, inhibition of RSK2 in PTEN-deleted neurons blocks the preconditioning effect: neurons grow shorter and highly ramified neurites, as in control condition without preconditioning (**Fig 5O–5R**). In parallel, we analyzed axon regeneration of sciatic nerve in these mice. We found that PTEN deletion does not significantly improve sciatic nerve regeneration, even if a trend is observed with the regeneration index (**S7K–S7M Fig**). When AAV8-Sh-RSK2 and AAV-Cre were injected in PTEN^f/f^ mice, we found that mTOR pathway activation does not counteract the inhibition of axon regeneration induced by RSK2 knockdown (**Fig 5S–5U**).

Altogether, our results show that RSK2 controls the preconditioning effect and PNS regeneration independently of mTOR.

### RSK2 controls the preconditioning effect via RPS6 phosphorylation

**Fig 6:**
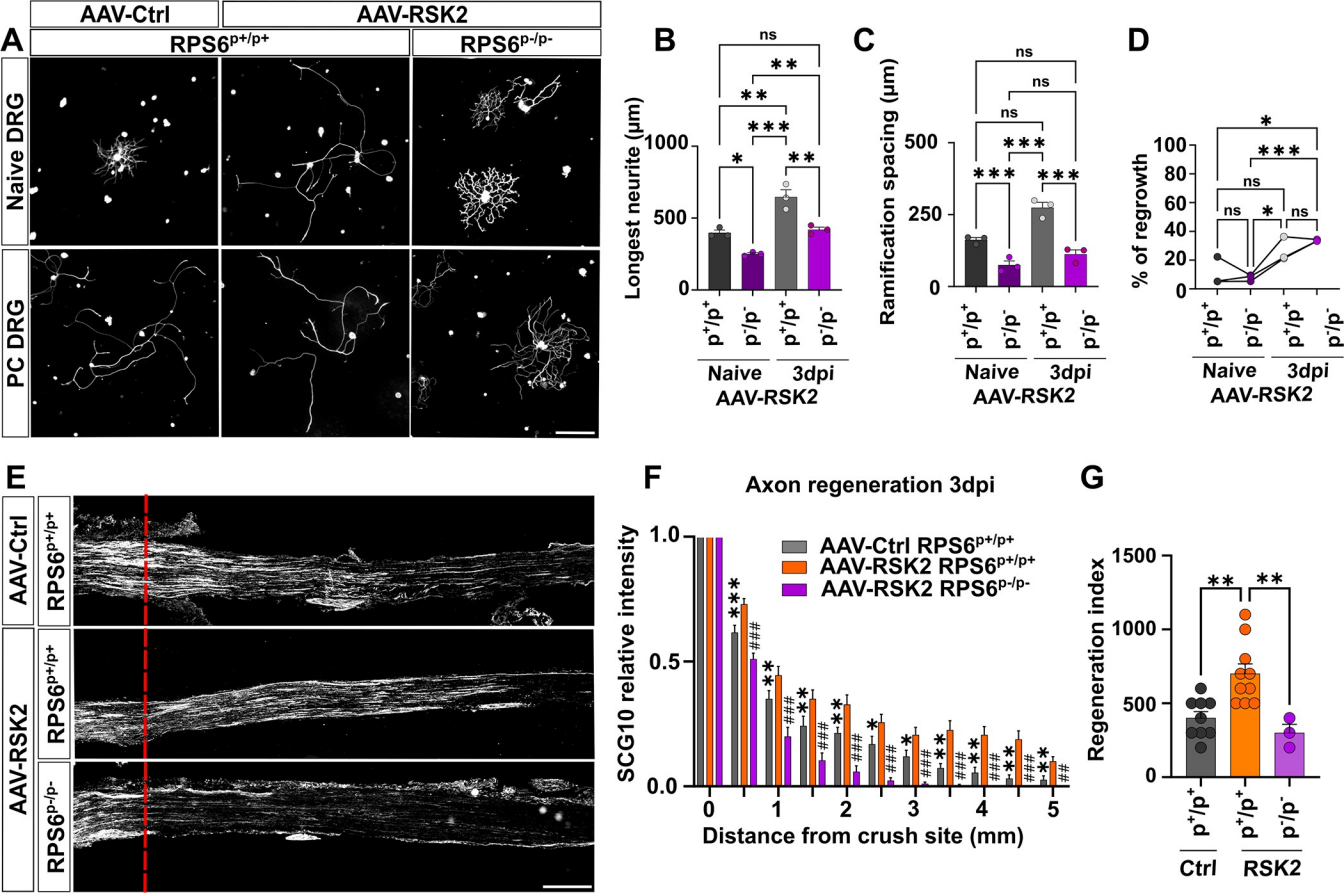
RSK2 needs a phosphorylable RPS6 to induce the preconditioning effect and axon regeneration in the PNS. (A) Representative microphotographs of DRG dissociated cultures showing that RSK2 overexpression phenocopies the preconditioning effect in naive cultures from RPS6^p+/p+^ mice but not from RPS6^p-/p-^ cultures. Scale bar: 250 μm. (B–D) Quantification of A. (B) Longest neurite length per neuron 16 h after plating (mean ± SEM, Ordinary one-way ANOVA, 3 independent DRG cultures, approximately 50 counted cells per condition per culture). (C) Mean distance between 2 ramifications (mean ± SEM, Ordinary one-way ANOVA, 3 independent DRG cultures, approximately 50 cells counted per condition per culture). (D) Percentage of neurons growing a neurite 16 h after plating (mean ± SEM, one-way ANOVA, 10 random microscopy fields quantified per condition). (E) Representative confocal images of the sciatic nerve sections 3 days post-injury from RPS6^p+/p+^ or RPS6^p-/p-^ mice injected intrathecally with AAV8-RSK2. Regenerating axons are labeled with anti-SCG10 antibody (white). The red dashed line indicates the injury site. Scale bar: 500 mm. (F) Quantification of regenerative axons from E (mean ± SEM, multiple *t* test, at least 3 animals per condition) (⁎, ⁎⁎, ⁎⁎⁎: comparison between groups AAV8-Ctrl in RPS6 ^p+/p+^ mice and AAV8-RSK2 RPS6 ^p+/p+^ mice; #, ##; ###: comparison between groups AAV8-RSK2 in RPS6 ^p+/p+^ mice and AAV8-RSK2 in RPS6 ^p-/p-^ mice). (G) Regeneration index at 3 dpi (mean ± SEM, unpaired *t* test, at least 3 animals per condition). ⁎⁎⁎*p* < 0.001, ⁎⁎*p* < 0.01, ⁎*p* < 0.05; ###*p* < 0.001, ##*p* < 0.01; #*p* < 0.05, ns: not significant. Raw data can be found in Supporting information (S1 Data). DRG, dorsal root ganglion; PNS, peripheral nervous system; RPS6, ribosomal protein S6.

Our results show that RSK2 regulates RPS6 phosphorylation. Moreover, RSK2 and p-RPS6 are both indispensable for the preconditioning effect (**Figs 2 and 5**). Thus, we asked whether RSK2 regulates the preconditioning effect via RPS6 phosphorylation. To do so, we overexpressed RSK2 in DRG of the unphosphorylable RPS6 mutant mouse line (**S1A Fig**), by injecting intrathecally AAV8-RSK2 or control in RPS6^p+/p+^ and RPS6^p-/p-^ 4-week-old mice. Three weeks later, we performed unilateral sciatic nerve injury. We first analyzed DRG cultures at 3 dpi. As expected, in naive RPS6^p+/p+^ DRG condition, RSK2 overexpression induces a preconditioning effect-like phenotype (**Fig 6A–6D**). Conversely, RSK2 overexpression in naive RPS6^p-/p-^ DRG does not lead to the preconditioning effect: neurites are short and highly ramified, as naive control cultures (**Fig 6A–6D**). Very interestingly, in preconditioned RPS6^p-/p-^ DRG cultures, RSK2 overexpression has no effect on neurons as they maintain their naive phenotype (**Fig 6A–6D**). Our results show that RSK2-mediated control of the preconditioning effect depends on RPS6 phosphorylation. In parallel, analysis of regeneration in the injured sciatic nerve showed that, in contrast to RPS6^p+/p+^ mice, overexpression of RSK2 in RPS6^p-/p-^ mice completely switches off axon regeneration (**Fig 6E–6G**). Altogether, our results show that the RSK2-RPS6 axis is critical for the preconditioning effect and peripheral nervous system regeneration.

### RSK2 controls spinal cord regeneration and functional recovery

**Fig 7:**
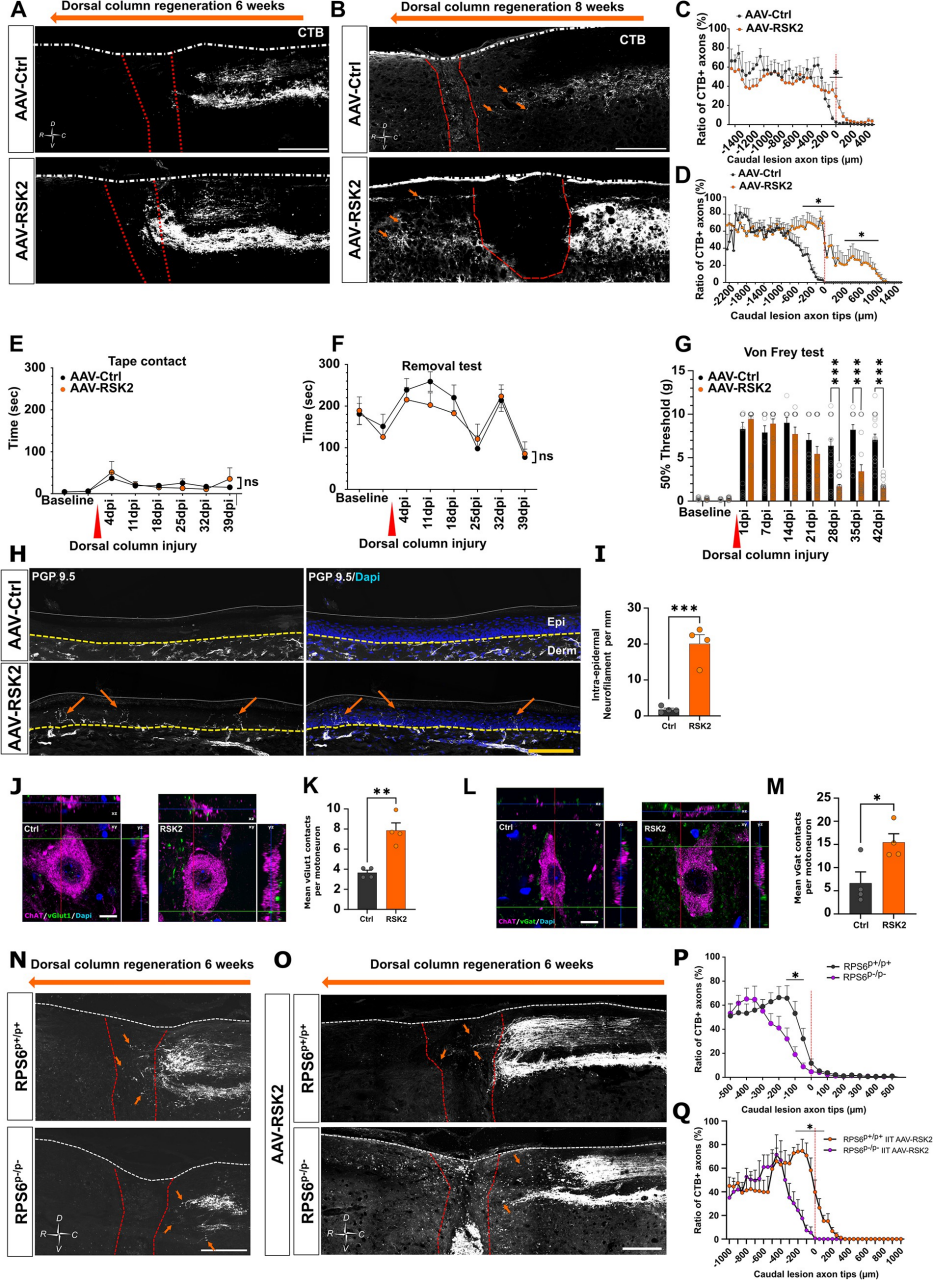
RSK2 induces dorsal column regeneration with functional sensory recovery. (A) Representative confocal images of thoracic spinal cord sagittal sections 6 weeks after dorsal column crush from mice injected intrathecally with AAV-Ctrl or AAV-RSK2. Regenerative axons are labeled with anti-CTB antibody (white). The orange arrow shows the direction of axon regeneration. (B) Representative confocal images of thoracic spinal cord sagittal section 8 weeks after dorsal column crush from mice injected intrathecally with AAV8-Ctrl or AAV8-RSK2. Regenerative axons are labeled with anti-CTB antibody (white). The orange arrow shows the direction of axon regeneration. (C) Quantification of axon regeneration and dieback from caudal marge of crush site from A (mean ± SEM, Mann–Whitney test, *N* = at least 6 animals per condition). (D) Quantification of axon regeneration and dieback from caudal marge of crush site from B (mean ± SEM, Mann–Whitney test, *N* = at least 4 animals per condition). (E, F) Tape contact and removal test in mice intrathecally injected with AAV8-Ctrl or AAV8-RSK2, 2 weeks before and 6 weeks after dorsal column crush (mean ± SEM, two-way ANOVA, *N*   = at least 11 animals per group, NS = non-significant). (G) Von Frey experiment to test nociception in mice injected intrathecally with AAV8-Ctrl or AAV8-RSK2 2 weeks before and 6 weeks after dorsal column crush; stimulus intensity is showed in grams (mean ± SEM, multiple *t* test, *N* = at least 11 animals per group; each paw was considered independently). (H) Sagittal section of glabrous skin of mice 15 days after sciatic nerve injury. Overexpression of RSK2, strongly increases the density of intra-epidermal neurofilament in contrast to control. (I) Quantification of intra-epidermal neurofilament staining per mm (scale bar: 100 μm). Regenerative fibers were labeled with anti-PGP 9.5 (neurofilament) antibody in white and nuclei with DAPI in blue (mean ± SEM, unpaired *t* test, 4 animals). (J) Multi-fluorescent orthogonal 3D confocal images showing the juxtaposition between vGlut1-positive boutons (green) and ChAT-positive motoneurons (magenta) below the injury site (L1-4) in sagittal sections of spinal cord 6 weeks after dorsal column injury. Scale bar: 10 μm. (K) Quantification of J (mean ± SEM, unpaired *t* test, 4 animals with at least 10 motoneurons quantified). (L) Multi-fluorescent orthogonal 3D confocal images showing the juxtaposition between vGat1-positive boutons (green) and ChAT-positive motoneurons (magenta) below the injury site (L1-4) in sagittal sections of spinal cord 6 weeks after dorsal column injury. Scale bar: 10 μm. (M) Quantification of L (mean ± SEM, unpaired *t* test, 4 animals with at least 10 motoneurons quantified). (N) Representative confocal images of thoracic spinal cord sagittal sections 6 weeks after sciatic nerve injury and dorsal column crush from RPS6^p+/p+^ or RPS6^p-/p-^ mice. Regenerative axons are labeled with anti-CTB antibody (white). In RPS6^p-/p^ mice, the preconditioning effect is inhibited in the spinal cord. (O) Representative confocal images of thoracic spinal cord sagittal sections 6 weeks after dorsal column crush from RPS6^p+/p+^ or RPS6^p-/p-^ mice intrathecally injected with AAV-RSK2. In RPS6^p-/p-^ mice, RSK2 overexpression fails to induce dorsal column regeneration in contrast to RPS6^p+/p+^ mice. (P) Quantification of axon regeneration and dieback from caudal marge of crush site from N (mean ± SEM, Mann–Whitney test, *N* = at least 4 animals per condition). (Q) Quantification of axon regeneration and dieback from caudal marge of crush site from O (mean ± SEM, Mann–Whitney test, *N* = at least 4 animals per condition). Raw data can be found in Supporting information (S1 Data). CTB, cholera toxin B; RPS6, ribosomal protein S6; vGAT, vesicular gamma aminobutyric acid transporter; Vglut1, vesicular glutamate transporter 1.

As the RSK2/RPS6 axis controls the preconditioning effect, we then asked whether it also controls CNS regeneration. To address this question, we focused on the sensory axons that form the dorsal column of the spinal cord. This bundle contains the central branch of the DRG. Like all CNS axons, these ones are unable to regenerate spontaneously after spinal cord injury [22,23]. Interestingly, the prior lesion of the sciatic nerve (the preconditioning paradigm) promotes axon regeneration in the spinal cord [22]. Since RSK2 controls the preconditioning effect via RPS6 phosphorylation, we asked whether RSK2 overexpression is sufficient to induce axon regeneration in the spinal cord. Thus, we injected intrathecally AAV8-RSK2 or AAV8-control in 4-week-old wild-type mice. Two weeks later, we performed dorsal column crush injury (**S8A Fig**). For each sample, analysis of cervical sections confirmed that the lesion was complete (**S8B Fig**). In control condition, axons reached the border of the lesion, with few axons observed within the injury site. No axon was found crossing the lesion area (**Fig 7A–7C**). When RSK2 is overexpressed in DRG (without the preconditioning paradigm), not only do axons enter the lesion site, but they also cross it and grow beyond the injury site. This phenotype is observed at 6 weeks post-injury and is exacerbated at 8 weeks post-injury (**Fig 7B–7D**).

We next assessed whether this regeneration can sustain functional recovery. To this end, we performed 2 behavioral assays to study sensitive function recovery: the tape contact and removal test (where first contact and total time for removal sticky paper was measured) and the Von Frey filament test. In the tape contact and removal test, we did not see any difference between control and RSK2 overexpression groups (**Fig 7E and 7F**). Interestingly, the Von Frey test revealed that mice overexpressing RSK2 have better functional recovery (**Fig 7G**). Indeed, immediately after dorsal column injury, we observed a loss of sensitivity in both groups. While this loss of sensory function was maintained in the control group throughout the whole experiment, the RSK2 overexpression group recovered sensitivity from 28 days after injury (**Fig 7G**). Together, our histological and behavioral analyses show that the RSK2 up-regulation induces CNS axon regeneration and functional recovery. We then addressed the underlying mechanisms of this functional recovery. We analyzed hindlimb paw skin innervation in control and upon RSK2 overexpression, 2 weeks after sciatic nerve injury (**Fig 7H and 7I**) as described in [32]. We found that overexpression of RSK2 promotes significantly skin innervation compared to control. Moreover, we found that RSK2 overexpression increases the number of vesicular glutamate transporter 1 (Vglut1) and vesicular gamma aminobutyric acid transporter (vGAT) positive boutons, respectively, excitatory and putative inhibitory synapses apposed to motoneurons labeled with ChAT (choline acetyl transferase) on the lumber spinal cord (**Fig 7J–7M**). These results suggest spinal circuit reorganization and synaptic plasticity between motoneurons and the propriospinal neurons. These results are in accordance with previously described mechanisms [33,34]. Altogether, our results show that RSK2 promotes functional recovery through enhanced axon regeneration and spinal cord plasticity.

To confirm these findings, we tested the effect of RSK2 inhibition on CNS regeneration after preconditioning. In this experiment, 4-week-old wild-type animals received an intrathecal injection of AAV8-ShRSK2 or control. Two weeks later, we performed unilateral sciatic nerve injury and the next day, we performed dorsal column crush injury. One week before sacrifice, we injected Alexa555-conjugated CTB into the sciatic nerve, upstream to the injury site (medial to the spinal cord), in order to assess dorsal column regeneration (**S8A Fig**). For each sample, analysis of cervical sections confirmed that the lesion was complete (**S8B Fig**). In control mice, regenerating axons reach the lesion site and some axons are able to cross it, as previously reported [22] (**S8C and S8D Fig**). When RSK2 is knocked down in DRG, despite the preconditioning paradigm, we observed a massive retraction of the axon bundle from the lesion site. No axon could reach the injury site (**S8C and S8D Fig**). In order to assess the effect of RSK2 inhibition on sensory functional recovery, we performed the same behavioral tests as described above. In both Von Frey test and the tape contact and removal test, inhibition of RSK2 significantly impairs functional recovery induced by the preconditioning (**S8E and S8F Fig**).

Finally, we assessed the dorsal column regeneration in the unphosphorylable RPS6 mutant mouse line, and 6-week-old RPS6^p+/p+^ and RPS6^p-/p-^ mice received unilateral sciatic nerve injury and 1 day after, dorsal column crush injury. One week before sacrifice, we injected Alexa555-conjugated CTB into the sciatic nerve, in order to assess dorsal column regeneration. Only animals with complete lesions were analyzed, as verified at the cervical level (**S8B Fig**). Regeneration was analyzed 6 weeks after spinal cord injury. As expected, in RPS6^p+/p+^ mice, the preconditioning lesion of the sciatic nerve induces regeneration in the dorsal column. In RPS6^p-/p-^ mice, on the other hand, this regenerative effect is completely abolished (**Fig 7N–7P**). This further confirms that RPS6 phosphorylation is essential to trigger axon regeneration in the dorsal column. As shown in **Fig 7A–7C**, RSK2 overexpression is sufficient to induce dorsal column regeneration without prior sciatic nerve injury. In order to address RSK2-RPS6 axis contribution to CNS regeneration, we overexpressed RSK2 in RPS6^p+/p+^ and RPS6^p-/p-^ mice. We performed the same experimental workflow as in Fig 7A. Our results show that the regenerative effect of RSK2 overexpression is blocked in RPS6^p-/p-^ mice (**Fig 7O and 7Q**).

Altogether, our results demonstrate that the RSK2/RSP6 axis is required for sensory axon regeneration in the spinal cord, synaptic plasticity, and target innervation, leading to functional recovery.

## Discussion

The current lack of efficient therapies for CNS regeneration remains a major challenge. Despite outstanding advances in the modulation of intrinsic regrowth capabilities in the past 15 years [1,8,9,35], full functional recovery has not been achieved yet. Rapidly, mTOR became a key pathway to trigger CNS regeneration [3,4]. However, not only the precise mechanisms by which mTOR leads to axon regeneration remain elusive, but also the exact contribution of one of its main effectors, the phosphorylated RPS6, is unknown. Moreover, during PNS regeneration and the preconditioning effect, mTOR has a modest contribution that can also depend on the experimental design used to assess its function [13,16,17]. These observations suggest that other pathways may be involved in these processes.

In our study, we demonstrate that RPS6 phosphorylation, in particular, on the Serine 235–236 is critical for PNS and CNS regeneration. We show that RPS6 phosphorylation is induced by sciatic nerve injury and is required for the preconditioning effect. In addition, we demonstrate that this phosphorylation is not controlled by mTOR but by the p90S6 kinase, RSK2. Moreover, RSK2 promotes regeneration of the central branch of DRG axons in the spinal cord, skin innervation, synaptic plasticity, and associated functional recovery. Altogether, our work sheds light on the critical role of RPS6 phosphorylation and on the importance of this posttranslational regulation by RSK2.

Increased RPS6 phosphorylation correlates with enhanced regenerative capacity, both in the CNS and the PNS [3,36]. Notably, RPS6 phosphorylation decreases in neurons during development and aging, which correlates with the decrease of regenerative ability [3,32]. In the mature retina, which belongs to the CNS, high levels of endogenous RPS6 phosphorylation are associated with better resilience and regenerative potential [10,37]. Other neurons like DRG neurons express endogenous phosphorylated RPS6, which further increases upon sciatic nerve injury. Our results show that even in DRG, neuronal subpopulations regulate differentially RPS6 phosphorylation. For some neurons such as the TrkB^+^ mechanosensitive neurons, axon lesion leads to increased RPS6 phosphorylation but not in others such as the itch-sensing Somatotstatin^+^ neurons. Interestingly, all these subpopulations regenerate their axon after injury. These results suggest that the basal endogenous level of phosphorylated RPS6 is an indicator of positive outcome regarding axon regeneration. Surprisingly, very few studies tackled the role of RPS6 posttranslational modifications—specifically phosphorylations—in the context of axon regeneration.

RPS6 is a ribosomal protein (RP) that belongs to the 40S subunit of the ribosome. It is one of the best studied RPs. Indeed RPS6 is the first RP for which inducible posttranslational modification has been reported upon liver injury [20]. The role of RPs during regulation of protein synthesis is still under debate. If we long thought that RPs were mostly required to ensure the structural integrity of the ribosome, several pieces of evidence tend to demonstrate that RPs directly control protein synthesis. Indeed, RPS6 phosphorylation has been proposed to regulate global protein synthesis, with a direct impact on translation initiation and elongation [28,38,39].

Interestingly, RPS6 phosphorylation has been shown to be important for ribosome biogenesis [40]. In particular, RPS6 is involved in the transcriptional regulation of Ribosome Biogenesis (RiBi) factors involved in pre-rRNA synthesis, cleavage, posttranscriptional modifications, ribosome assembly, and export. Therefore, one can hypothesize that increase of RPS6 phosphorylation promotes ribosome biogenesis and subsequent enrichment of the pool of ribosomes in cells. Axon regeneration requires extensive protein synthesis to generate all the building blocks to sustain axon growth, as illustrated by the level of global protein synthesis that is decreased in neurons upon axon injury [3]. Thus, increasing the number of ribosomes may help to sustain high levels of protein synthesis to support axon regeneration.

Furthermore, Bohlen and colleagues have demonstrated that the level of RPS6 phosphorylation differentially affects mRNA translation based on the length of their coding sequence (CDS) [41]. As ribosomes translate mRNAs, RPS6 are progressively dephosphorylated. Therefore, mRNA with short CDS are actively translated by phosphorylated RPS6. Interestingly, many genes involved in axon regeneration have short CDS and can be preferentially translated by ribosomes with phosphorylated RPS6 [41], such as ATF3 [42] (ATF3_MOUSE 181 aa), KLF family [43] (KLF7_MOUSE 301 aa), Rheb [35] (RHEB_MOUSE 184 aa), or genes implicated in mitochondria function [44] (PPARG_MOUSE 505aa, UCP2_MOUSE 309 aa, ARMX1_MOUSE 456 aa). Based on these observations, RPS6 phosphorylation may prime neurons for regeneration by facilitating the translation of pro-regenerative mRNAs.

We show that RSK2 controls RPS6 phosphorylation, which in turn controls the preconditioning effect and axon regeneration both in the CNS and the PNS. RSK acts downstream of the MAPK pathway as it is activated by ERK1/2. The RSK family is closely related to the MSK (mitogen and stress activated kinase—MSK1 and 2) family. However, their physiological functions are different [45]. RSK have 2 kinase domains. The N-terminal kinase domain is an AGC family kinase that shares 57% of amino acids with the S6K1 kinase domain. The C-terminal kinase domain is related to the CAM-K kinase family. Thus, despite potential sharing of substrates with S6K1, RSK may have specific targets. RSK promotes the phosphorylation of RPS6 on Ser235-236, which in turn promotes the assembly of the translation complex. This process correlates with an increase of CAP-dependent translation, independently of the mTOR pathway [46]. Our results show that RSK2/RPS6 phosphorylation-controls regeneration independently of mTOR activation. In fact, this suggests that mTOR and RSK2 will have different regenerative outcomes, possibly depending on the neuronal subpopulation. Importantly, in DRG, mTOR and RSK pathways are not redundant and they do not compensate each other.

In this study, we focused on the RSK-RPS6 axis, yet RSK is known to phosphorylate several other substrates that could participate in axon regeneration. For example, RSK2 controls the transcription regulation of c-fos [47] and CREB [48], which are both involved in axon regeneration [49]. RSK2 is also important in the process of cell growth based on its regulation of GSK3β phosphorylation that has been described to promote CNS regeneration [50,51]. Finally, RSK2 positively regulates cell survival [52]. As neuronal survival is key for the outcome of injury response, RSK2 may be implicated in the neuroprotection observed after sciatic nerve lesion. Altogether, a larger analysis of the diverse phosphorylated targets of RSK2 in DRG neurons and in CNS neurons will give us more insight into the precise mechanisms of RSK2-dependent regeneration.

Interestingly, a study from Mao and colleagues also identified RSK family as key effectors of PNS and CNS regeneration [27]. They found that RSK1 contributes to sciatic nerve regeneration. Mechanistically, authors described that overexpression of the elongation factor eEF2 rescues the effect of RSK1 inhibition both in vitro and in vivo. They showed that eEF2 controls the translation of pro-regenerative mRNAs such as BDNF or IGF, which have been largely described to modulate axon regeneration and neuroprotection [10,53]. Both molecules partially rescue the deletion of RSK1 in vitro. Interestingly, based on their study and ours, RSK1 and RSK2 seem to have a similar pro-regenerative effect in the PNS. However, while both mechanisms of action are based on translational control, the modalities and effectors are different. eEF2 factor is a canonical translational factor implicated in the regulation of translation elongation. RSK1-mediated phosphorylation of eEF2 kinase promotes translation that is necessary for regeneration. Both Mao and colleagues’ work and ours demonstrate that RSK family critically regulates the posttranslational modification of components of the translational complex, thereby controlling protein synthesis and axon regeneration. To note, RSK2 can phosphorylate eEF2K and RSK1 can also phosphorylate RPS6. It would be interesting to decipher if RSK1 and 2 co-expression synergies to further enhance axon regeneration.

Mao and colleagues also addressed the role of RSK1 in CNS regeneration by using the visual system. They showed that RSK1 overexpression in RGC has no effect on regeneration nor on neuroprotection. This contrasts with our data showing that RSK2 promotes CNS regeneration in the dorsal column. Two hypotheses can explain this discrepancy. First, even if RSK1 and 2 share common targets and are often implicated in the same biological functions [26], they also have their own specific substrates leading to the specificity of action of each isoform. Second, there may be a cell type specificity of RSK family function. Even if a large spectrum of neuroprotective and regenerative molecular pathways is shared between the different CNS and PNS neuronal populations, neurons have cell type- and subpopulation-specific injury responses. This feature has been illustrated by the sc-RNAseq analysis of single RGC that uncovered intrinsically resilient subpopulations of RGC [54,55]. This suggests that each subpopulation of neurons has an intrinsic specific machinery that influences its response to stress. In our case, the regenerative effect of RSK2 in other CNS regeneration models remains to be determined. Nonetheless, we can propose that DRG are more prompt to respond to RSK activity compared to RGC. Interestingly, PTEN deletion-induced activation of mTOR combined with RSK1 overexpression leads to a synergistic effect on optic nerve regeneration, with axons reaching the distal part of the optic nerve [27]. In DRG, we found that RSK2-mediated phosphorylation of RPS6 is mTOR independent, whereas in RGC, mTOR may be required to phosphorylate RPS6, along with RSK1-mediated control of eEF2 activity. Altogether, mTOR-RSK interactions may well depend on the neuron type in order to control RPS6 phosphorylation.

To conclude, our work demonstrates that RPS6 phosphorylation is key in the process of PNS and CNS regeneration. Its regulation by RSK2 independently of mTOR highlights the role of this pathway in regeneration and opens new avenues to understand molecular mechanisms of axon regrowth and functional recovery.

## Material and methods

### Surgical procedures

Animal care and procedures were performed according to the Grenoble Institute Neurosciences, French (French Ministry of research guidelines) and European guidelines (directive 86/609 and 2010/63) (APAFIS #38155–202205021448189 v5).

For intrathecal injections and dorsal column crush, mice were anesthetized with a mix of ketamine (100 mg/kg) and xylazine (10 mg/kg). Sciatic nerve crush procedure was performed under 3% induction, 2% maintenance isoflurane. For analgesia, paracetamol was given in the drinking water (4 mg/ml) 1 day before and 2 days after surgery. Buprenorphine (0.05 mg/kg) was administered by subcutaneous injection 6 h before dorsal column injury and every 6 h for 3 days after surgery.

### Animals

Mice with mixed backgrounds were used as wild-type animals, regardless of their sex. Phospho-dead RPS6 mouse line has been already described [28] and is maintained in mixed background. Animals were kept on a 12 h light/dark cycle with food and water provided at libitum, at a constant temperature and humidity (21°C; 10% humidity). In all experiments, mice showing any signs of hindlimb paralysis or any discomfort were removed from further experiments.

### AAV8 virus injections

The 3 to 4 weeks old mice were injected intrathecally, as described previously [56] using a 30G needle with 10 μl of the following viruses: AAV8-PLAP (placental alkaline phosphatase; as control), AAV8-GFP (as control), AAV8-CRE, AAV8-RSK2, AAV8-RSK3, AAV8-shScrambled, AAV8-shRSK2, AAV8-RPS6^235D-236D^, or AAV8- RPS6^240D-244D-247D^. Virus titers were around 1 × 10^14 particles/ml.

For co-infections, 10 μl of a mix of viruses was injected.

### Sciatic nerve crush

The left sciatic nerve was exposed and crushed for 15 s with forceps (Fine Science Tools, Dumont SS Forceps) with an angle of 45°. The sciatic nerve was crushed again at the same place for 5 s to ensure that all axons have been axotomized.

### Dorsal column injury

The 5 to 6 weeks old mice underwent laminectomy at the level of T7 vertebra exposing the spinal cord. Using modified fine forceps (Fine Science Tools; Dumont #5SF Forceps; maximum amplitude of 1 mm, adjusted with a rubber), the dorsal column was crushed 3 × 10 s with 700 μm of depth.

### Dorsal column retrograde labeling

After exposing the injured sciatic nerve, 3 μl of CTB (Cholera Toxin Subunit B, Alexa Fluor 555 Conjugate—1 mg/ml—Thermo Fisher) was injected in sciatic nerve upper to the lesion site with a glass micropipette to analyze the extend of dorsal column regeneration in the spinal cord.

### Adult DRG neurons culture, drug treatment, and immunostaining

DRG cultures were performed as describes previously [57]. DRG from WT or animals that underwent intrathecal injection of AAV8 3 weeks before were collected. Briefly, lumbar DRG (L3-L4 and L5) were dissected out and collected in iced cold Hank’s balanced salt solution (HBSS; Gibco). DRG tissues were incubated in 5% Collagenase A (Roche) at 37°C for 70 min and in 0.25% Trypsin (Gibco) for 5 min. DRG were gently dissociated with blunt glass pipettes. Neurons were purified on a 10% bovine serum albumin (Sigma Aldrich) cushion and plated on Poly-L-lysine (10 mg/ml, Sigma Aldrich) and Laminin (0.5 mg/ml, Sigma Aldrich)-coated cover slips in Neurobasal A medium (Gibco) supplemented with 2% B-27 supplement (Gibco) and 1% L-glutamine (Gibco). Cultures were maintained in a humidified atmosphere at 5% CO2 in air at 37°C.

In case of drug treatment, neurons were treated 1 h after plating with RSK inhibitor BRD7389 3 μm (Santa Cruz), mTOR inhibitors Torin1 5 nM (Santa Cruz) or Rapamycin 0.1 nM (Sigma Aldrich), S6K1 inhibitor PF-4708671 8 μm (Sigma Aldrich), and Translation inhibitor Cycloheximide 2 nM (Sigma Aldrich). All the dilutions were performed in DMSO. For each group treated with drugs, the respective control received DMSO treatment.

After 16 h of culture, neurons were fixed in PFA 4%/sucrose 1.5% in PBS, permeabilized in 0.5% Triton (Sigma Aldrich), and labeled with primary antibodies, anti-Tuj1 (1/500, Biolegend), anti-pS6^Ser235-236^ (Ser 235–236; 1/500-Cell signaling Technology), anti-pS6^Ser240-244^ (Ser 240–244; 1/500-Cell Signaling Technology), anti-RSK (1/500, RSK123-Cell Signaling Technology), anti-His (1/500; ProteinTech), anti-Flag (1/500, Sigma), anti-V5 (1/200, ProteinTech), anti-PGP9.5 (1/500, Proteintech), anti-NeuN (1/500, Abcam), anti-alpha-tubulin (1/500, Thermo Fisher) for 2 h at room temperature and secondary antibodies, Alexa-488, Alexa-647, and Alexa-568 (1/800-Thermo Fisher) for 1 h at room temperature. Coverslips were mounted with Fluoromount-G Mounting Medium, with DAPI (Invitrogen).

### Cell transfection and protein extraction

N2A cells were transfected using PEI (3 mg/ml; Alfa Aesar-Thermo Fisher) with 3 μg of the following plasmids: pAAV-vsvg-RSK1, pAAV-flag-RSK2, pAAV-v5-RSK3, pAAV-his-RSK4, pAAV-PLAP, pAAV-CMV-mCherry-U6-sgRNA_shRSK2 (5′ GGACCAACTACCACAATACCA 3′), pAAV-CMV-mCherry-U6-sgRNA_shCtrl (5′ GCAACAACGCCACCATAAACT 3′). These plasmids were obtained by cloning cDNA extracted from mouse cerebellum in pAAV-MCS Expression Vector with In-Fusion Cloning system (Takara) and pAAV-RPS6^235D-236D^; AAV8-RPS6^240D-244D-247D^. These plasmids were obtained by cloning cDNA extracted from brain of RPS6^p-/p-^ mouse in pAAV-MCS Expression Vector with In-Fusion Cloning system (Takara) and specific point mutations were carried out by targeted mutagenesis. Approximately 96 h after transfection, proteins were extracted using RIPA (Triton 1%) buffer supplemented with protease (Roche) and phosphatase inhibitors (Roche).

For DRG, proteins were extracted using 10 mM Tris-HCl (pH 7.5); 150 mM NaCl; 0.5 mM EDTA; 1% NP-40 with protease and phosphatase inhibitors (Roche). DRG were further lysate by sonication (Vibra-Cell, VWR) 5 times, 10 s.

Proteins were quantified with BCA following manufacturer’s instructions (Pierce-Thermo Fisher).

### Ribosome purification

Ribosome purification was performed from N2A cells as previously described [58]. Briefly, cells were lysed in an appropriate hypotonic buffer with 0.7% of NP-40. After centrifugation at 750 g, the nuclear fraction was removed. The cytoplasmic fraction was submitted to centrifugation at 12,500 g to remove the mitochondrial fraction. The KCl concentration of the post-mitochondrial fraction was then adjusted to 0.5 M. Finally, ribosomes were purified using a sucrose cushion by ultracentrifugation at 240,000 g. Ribosome pellets were resuspended and the concentration of ribosome was estimated with DO_260_ RNA absorbance on a Nanodrop reader.

### Western blot

Approximately 20 μg of proteins from N2A cells or 10 μg of protein from DRG were loaded to 4% to 15% SDS-polyacrylamide precast gels (Biorad) and transferred to nitrocellulose membranes. Membranes were stained with Ponceau Red to verify the quality of the transfer. Membranes were then blocked in 5% low fat milk for 1 h in Tris buffered saline with 0.05% of Tween-20 (TBST) for 1 h at room temperature and incubated overnight at 4°C with the following antibodies diluted in blocking solution: anti-RSK2 (1/1,000, Cell Signaling Technology), anti-RPS6 (1/1,000, Cell Signaling Technology), anti-phospho-RPS6 ser235/236 (1/1,000, Cell Signaling Technology), anti-phospho-RPS6 ser240/244 (1/1,000, Cell Signaling Technology), anti-Flag (1/2,000, Sigma Aldrich), Anti-His (1/5,000, Proteintech), anti-RFP (1/1,000, Abcam), anti-GFP (1/1,000, Abcam), anti-GAPDH (1/5,000, Proteintech), anti-TOMM40 (1/500, Proteintech), and anti-H3 (1/2,000, Cell Signaling Technology). Membranes were then incubated for 2 h at room temperature with HRP-coupled secondary antibodies (anti-rabbit HRP, Proteintech; anti-mouse HRP, Thermo Fisher Scientific, anti-chicken HRP Thermo Fisher Scientific) diluted from 1/1,000 to 1/10,000 in blocking solution. Membranes were developed with ECL (1.5 mM luminol, 0.225 mM coumaric acid, 100 mM Tris HCl, 0.1 mM hydrogen peroxide in milliQ water) using a chemidoc (ChemiDoc MP, Biorad).

### Histological procedures

After intracardial perfusion of mice with ice cold PFA-4%, tissues were dissected out and post fixed in 4% PFA for 2 h for sciatic nerve and DRG, 24 h for spinal cords at 4°C. After cryopreservation in 30% sucrose, tissues were sectioned using a cryostat: DRG and sciatic nerves were sectioned at 12 μm and spinal cords at 20 μm. For skin hind paw, mice hair was first removed and then mice were perfused intracardiacally with ice cold PBS. The hind paw skin was dissected out and post fixed 24 h in PFA 4% in PBS. The skin was bleached in 5% H2O2/PBS solution overnight. After cryopreservation in 30% sucrose, 20 μm sagittal skin cryosections were performed.

### Immunohistochemistry

Sections were incubated in blocking solution (5% BSA, 1% Donkey Serum, and 0.5% Triton in DPBS) for at least 1 h at room temperature. Sections were then incubated overnight at 4°C with primary antibodies diluted in blocking solution: anti-Tuj1 (1/500, Biolegend), anti-RSK2 (1/100, Cell Signaling Technology), anti-RPS6 (1/100, Cell Signaling Technology), anti-p-RPS6^Ser235-236^ (1/100, Cell Signaling Technology), anti-p-RPS6^Ser240-244^ (1/500, Cell Signaling Technology), anti-RFP (1/500, Abcam), anti-SCG10 (1/1,000, Novus), anti-CTB (1/500, Abcam), anti-vGlut1 (1/1,000, Synaptic System), anti-vGat (1/500, Synaptic System), anti-ChAT (1/100, Merck), anti-Islet1-2 (5 μg/ml, DSHB), anti-Advillin (1/500, Proteintech), anti-TrkA (1/500, Bio-techne), anti-Parvalbumine (1/200, Swant), anti-TrkB (1/500, Bio-techne), anti-Calbindin (1/100, Swant), anti-Somatostatin (1/500, Invitrogen), and anti-PGP 9.5 (1/500, Proteintech). Then, tissues were incubated with the appropriated secondary antibodies (Alexa-Fluor conjugated—Jackson laboratories) diluted in blocking solution at 1/500 for 2 h at room temperature. Slides were mounted with Fluoromount-G Mounting Medium, with DAPI Medium (Invitrogen).

### In situ hybridization

Experiments were performed as described in Nawabi and colleagues [59]. Probes were cloned in pGEMT vector from cDNA extracted from cerebellum: pGEM-T_RNAprobeRSK1; pGEM-T_RNAprobeRSK2; pGEM-T_RNAprobeRSK3; pGEM-T_RNAprobeRSK4 Sequence used for the probe was described in S1 Table.

### Image analysis and quantification

DRG cultures, DRG sections, and sciatic nerves sections were imaged using a Nikon Ti2 Eclipse epifluorescent microscope with 4×, 10×, and 20× objectives. Spinal cord sections and hind paw sections were imaged using a Dragonfly spinning disk microscope (Andor Technologies) with a 20× or 63× objective. Sections were stitched using the Fusion software with 10% overlap between tiles. Synaptic contacts to motoneuron were imaged with a Confocal LSM710/Airyscan (Zeiss) with a 63× objective. Multi-fluorescent orthogonal 3D image analysis and visualization were performed using Zen 3.2 software.

### Analysis of neurite outgrowth, ramification, and survival

The mean of neurite outgrowth, ramification, and survival of DRG neurons was manually measured with ImageJ software. The mean neurite outgrowth for at least 50 neurons per condition (except for BRD7389 and cycloheximide condition) was quantified for at least 3 independent biological replicates. Neurite ramification was analyzed for at least 30 neurons per condition from at least 3 independent biological replicates. DRG neurons survival was quantified from 10 random microscope fields per condition from at least 3 independent biological replicates.

### Analysis of sciatic nerve regeneration

Axon regeneration was quantified on 3 to 5 sagittal sections for each mouse. SCG10 intensity was quantified with ImageJ software in 500 μm area, the background intensity was subtracted, and residual intensity was compared to the crushed area (maximum intensity of SCG10). Regeneration index was determined by measuring the distance from the crush site to the location where SCG10 intensity is the half of the intensity at the crush site.

### Analysis of skin re-innervation

Approximately 15 days after sciatic nerve crush, mice were anesthetized and its hind paw was depilated with cream. Mice were intracardiacally perfused with cold ice PBS and glabrous skin was gently removed from bones. After processing, skin re-innervation was quantified in 3 to 4 mm of glabrous skin hind paw per animal. Number of fibers in epidermis was quantified with ImageJ software.

### Analysis of dorsal column regeneration

Axon regeneration was quantified on 2 to 4 sections for each mouse. CTB intensity was quantified with ImageJ software every 50 μm, the background intensity was subtracted, and residual intensity was compared to maximum intensity. The origin of regeneration/dieback (distance “0”) is the caudal part of the crush site defined by the borders of the glia scar.

### Analysis of vGlut1 and VGAT synaptic boutons

VGlut1 and VGAT synaptic boutons were imaged with a Confocal LSM710/Airyscan (Zeiss). Z-stack images were taken with an average thickness of 18 μm with a step size of 0.2 μm. Multi-fluorescent orthogonal 3D image analysis and visualization were performed using Zen 3.2 software. The average number of vGlut1 or VGAT boutons opposed to motor neurons from L1-4 spinal sections was calculated by analyzing at least 10 motoneurons per sample.

### Analysis of fluorescence intensity in DRG cells

For quantitative analysis of fluorescence intensity (p-S6^Ser235-236^; p-S6^Ser240-244^, RSK2, Isl1/2, Advillin, TrkA, Parvalbumin, TrkB, Calbindin, and Somatostatin), DRG neurons and nuclei were manually outlined in ImageJ software, only cytoplasmic pixel intensity was quantified. For each marker, the setting was fixed for all acquisitions.

### Analysis of shRNA effect on RSK2 expression

For quantitative analysis of RSK2 fluorescence intensity, DRG neurons and nuclei were manually outlined in ImageJ software, only cytoplasmic pixel intensity was quantified. To analyze the effect of shRNA-RSK2, the expression of RSK2 was quantified and compared in mCherry positive DRG (infected neurons) and mCherry negative DRG neuron (uninfected neuron) from the same section. These data were compared to sh-Scrambled effect also reported to the intensity of RFP protein. For each experiment, imaging settings were fixed for all acquisitions.

### Behavior tests

For behavior tests, we used mix background, male and female mice from pooled litters. Before the first surgery (intrathecal injection), mice were handled once a day with soft and strong contention, head belly, and foot contact. After the first surgery, for the Von Frey filament, mice were placed 10 min per day during 7 days in a 10-cm diameter glass ramekin on non-sharpness grid at 60 cm above the floor. For the removal of the sticky paper, mice were placed on individual cages and trained 7 days on active phase with the sticky paper stuck in both paws until they were able to remove the sticker. After training, all experiments were performed once a week, 2 weeks before dorsal column injury and 6 after.

### Tape contact and tape removal test

For this test, mice were placed in the experiment room at least 1 h before the behavior test, and the experiment was performed during the activity period of mice (during night) with red light only. Mice were placed in a transparent “look-like” home cage. After at least 5 min of acclimatization, an 8-mm diameter adhesive pad was stuck to each hind paw. Time of first contact between mice nose and the sticky paper and the time needed for its removal was quantified for each hind paw. This experiment was performed twice each time with a maximum given time of 5 min to remove both pads [33]. Mice activity was recorded with 2 cameras (Logitech HD 1080p/60 fps) for further analyses.

### Von Frey filament test

For this test, each animal was placed in the experiment room at least 1 h before the behavior assay. Each mouse was individually placed in a 10-cm diameter bottomless box 10 min before the test. The box was placed on non-sharpness grid 60 cm above the floor. The method used in this test was the up and down method as described previously [60]. Briefly, once mice had calm down, they were tested for 3 s with the reference filament in the center of the paw. In case of reaction, the next test was performed with smaller filament (more sensitive). If the mice did not respond, the next test was performed with a thicker filament (less sensitive). To determine mice sensitivity, they had to respond 3 times for the same filament. This experiment was done for both paws independently. Mice activity was recorded with camera (Logitech HD 1080p/60 fps) for further analyses.

### Statistical analysis

All animals used were both male and females from pooled litters and were randomly assigned to groups before any treatment or experimental manipulation. All analyses were performed while blinded to the control test realized at the same time. Statistical analysis was performed with GraphPad Prism 9.4 using either one-sample *t* test, one-way ANOVA, two-way ANOVA, Kruskal–Wallis test, paired *t* test, unpaired *t* test. Each test used is indicated in figure legends. Error bars indicate the standard error of the mean (SEM). A *p*-value *p* < 0.05 was considered statistically significant with difference indicate by stars: ⁎⁎⁎*p* < 0.001, ⁎⁎*p* < 0.01, ⁎*p* < 0.05. ns: not significant.

## Supporting information

S1 Fig RPS6 phosphorylation level in different subpopulations of DRG neurons and their regenerative ability. (A) Schematic illustration of sensory neuron subtypes in adult DRG, based on their functions and the markers they expressed: TrkA, TrkB, Calbindin, Somatostatin, and Parvalbumin. (B) Representative microphotographs of DRG sections stained with anti-p-S6^Ser235-236^ (in magenta), CTB (in gray, only at 3dpi), and different DRG subpopulations markers (in green) in intact and 3dpc. Scale bar: 50 μm. (C) Graphs showing the quantification of B with a differential up-regulation of p-S6^Ser235-236^ at 3-dpi among different DRG subpopulations (mean ± SEM; unpaired *t* test; *N* = 3 animals per group; at least 50 positive neurons for each marker were counted). (D) Graphs showing proportion of CTB retro-labeled subpopulations in intact DRG and their proportion 3 dpi (Chi-squared test; at least 37 positive neurons for each marker were counted). Raw data can be found in Supporting information (S1 Data).
(TIF)

S2 Fig Characterization of phospho-dead RSP6 mouse line. (A) Schematic describing the unphosphorylable RSP6 mouse line. (B) Representative microphotographs of DRG sections from RPS6^p+/p+^, RPS6p^+/p-^, and RPS6^p-/p-^ stained with anti-RPS6, anti-p-S6^Ser235-236^, or anti-p-S6^Ser240-244^ (in magenta) and anti-Tuj 1 (in gray). Scale bar: 25 μm. (C) Western blot showing that RPS6 is not phosphorylated in RPS6^p-/p-^ DRG compared to RPS6^p+/p+^ and RPS6^p-/p+^ DRG. (D, E) Quantification of C (mean ± SEM; Ordinary one-way ANOVA; *N* = 3 animals per group). (F) Representative microphotographs of naive cultures of mature DRG neurons from WT (RPS6^p+/p+^) and homozygous (RPS6^p-/p-^) mice line defective for RPS6 phosphorylation showing no differences. Scale bar: 250 μm. (G–I) Graphs showing the quantification of F. (G) Longest neurite length per neuron 16 h after plating (mean ± SEM, unpaired *t* test, 3 independent DRG cultures, approximately 50 cells counted per conditions per culture). (H) Distance between 2 ramifications in longest neurite (mean ± SEM, unpaired *t* test, 3 independent DRG cultures, approximately 50 cells analyzed per condition per culture). (I) Percentage of neurons growing a neurite 16 h after plating (mean ± SEM, unpaired *t* test, 10 random microscopy fields quantified per condition, ns: non-significant). Raw data can be found in Supporting information (S1 Data and S1 Raw Images).
(TIF)

S3 Fig Overexpression of phosphomimic RPS6^235D-236D^ induces the preconditioning effect in naive DRG and has a modest effect on sciatic nerve regeneration in WT mice. (A, B) Western blot of ribosome purification showing a good integration of phosphomimetics RPS6 constructs (A) RPS6 ^240D-244D-247D^ or (B) RPS6^235D-236D^ in ribosome of N2A cells. (C) Representative microphotographs of naive cultures of mature DRG neurons from WT mice 21 days after intrathecal injection of AAV8-Ctrl; AAV8-RPS6^240D-244D-247D^ or AAV8-RPS6^235D-236D^ showing that only overexpression of AAV8-RPS6^235D-236D^ induces the preconditioning effect. Scale bar: 250 μm. (D, E) Graphs showing the quantification of C. (D) Longest neurite length per neuron 16 h after plating (mean ± SEM, one-way ANOVA, 3 independent DRG cultures, approximately 50 cells counted per condition per culture). (E) Distance between 2 ramifications in longest neurite (mean ± SEM; one-way ANOVA, 3 independent DRG cultures, approximately 50 cells analyzed per condition per culture). (F) Percentage of neurons growing a neurite 16 h after plating (mean ± SEM, two-way ANOVA, 10 random microscopy fields were quantified per condition). (G) Representative confocal images of sciatic nerve sections 3 days post-injury from WT mice injected intrathecally with AAV8-PLAP (control), AAV8-RPS6^240D-244D-247D^, or AAV8-RPS6^235D-236D^. Regenerating axons are labeled with anti-SCG10 antibody (white). The red dashed line indicates the injury site. Scale bar: 500 μm. (H) Quantification of regenerative axons from G (mean ± SEM, two-way ANOVA, at least 5 animals per group). (I) Regeneration index at 3 dpi (mean ± SEM, one-way ANOVA, at least 5 animals per group). (J) Representative microphotographs of mature naive DRG neurons cultures from RPS6^p-/p-^ mice, 21 days after intrathecal injection of AAV8-Ctrl; AAV8- RPS6^240D-244D-247D^ or AAV8-RPS6^235D-236D^ showing that only overexpression of phosphomimic AAV8-RPS6^235D-236D^ induces the preconditioning effect. Scale bar: 250 μm. (K–M) Graphs showing the quantification of J. (K) Longest neurite length per neuron 16 h after plating (mean ± SEM, one-way ANOVA, 3 independent DRG cultures, approximately 50 cells counted per condition per culture). (L) Distance between 2 ramifications in longest neurite (mean ± SEM, one-way ANOVA, 3 independent DRG cultures, approximately 50 cells analyzed per condition per culture). (M) Percentage of neurons growing a neurite 16 h after plating (mean ± SEM, two-way ANOVA, 10 random microscopy fields quantified per condition). ⁎⁎⁎*p* < 0.001, ⁎⁎*p* < 0.01, ⁎*p* < 0.05, ns: non-significant. Raw data can be found in Supporting information (S1 Data and S1 Raw Images).
(TIF)

S4 Fig Characterization of the effect of different signaling pathways on naive DRG cultures. (A) Ribosomal S6 kinase schematic signaling pathway and inhibitors (in red) used in this study. (B) Representative microphotographs of naive DRG neurons cultures treated with DMSO (control), a global protein translation inhibitor (cycloheximide (5 nM)); mTOR inhibitors (Torin1 (5 nM) or Rapamycin (0.1 nM)); and an S6K1 inhibitor (PF-4708671 (8 μm)). Scale bar: 250 μm. (C) Quantification of B (mean ± SEM, two-way ANOVA, 3–4 independent DRG cultures, approximately 50–100 cells counted per condition per culture (except for cycloheximide)). (D) Percentage of neurons growing a neurite 16 h after plating from A (mean, two-way ANOVA, 3–4 independent DRG cultures, 10 random microscopy fields were quantified per condition). ⁎⁎⁎*p* < 0.001, ⁎⁎*p* < 0.01, ⁎*p* < 0.05, ns: not significant. Raw data can be found in Supporting information (S1 Data).
(TIF)

S5 Fig Characterization of RSK family expression in mature DRG. (A) Graph showing the homology of amino acid sequences among the 4 RSK expressed in mouse. (B) Table summarizing the homology and identity among RSK1, 2, 3, and 4. (C) Schematic of the probes used to study specific expression of RSK1, 2, 3, and 4 by in situ hybridization. (D) Microphotographs showing in situ hybridization with sense and anti-sense RNA probes of RSK1, RSK2, RSK3 on adult brain coronal sections and RSK4 on embryonic E12.5 sagittal section showing specificity of these probes. (E) Workflow of experiment. (F) Microphotographs showing in situ hybridization of RSK1, RSK2, RSK3, and RSK4 on adult lumbar DRG sections in intact and after sciatic injury at 1, 3, and 7 days post-injury (dpi). Only RSK2 and RSK3 are highly expressed in mouse lumbar DRG and RSK2 expression is regulated by axon injury. Scale bar: 50 μm.
(TIF)

S6 Fig Characterization of ShRNA-RSK2. (A) Schematic of the plasmid constructs used to overexpress RSK1-VSVG, RSK2-Flag, RSK3-V5, RSK4-His, PLAP, or shRNA (sh-Scrambled or sh-RSK2). (B–E) Western blot showing shRNA-RSK2 specificity in N2A cells 96 h after co-transfection (mean ± SEM, one-sample *t* test, *N* = 3 transfections per group). (F) Representative microphotographs of DRG sections stained with anti-RFP (in magenta) and anti-Tuj 1 (in gray) antibodies, 21 days after intrathecal injection of AAV8-shCtrl (that co expressed the RFP). Scale bar: 25 μm. (G) Quantification of H (mean ± SEM, 3 animals, 5 DRG sections counted per animal). ⁎⁎⁎*p* < 0.001, ⁎⁎*p* < 0.01, ⁎*p* < 0.05. Raw data can be found in Supporting information (S1 Data and S1 Raw Images).
(TIF)

S7 Fig RSK3 is not involved in the preconditioning effect but PTEN deletion leads to a modest enhancement of sciatic nerve regeneration. (A) Workflow of experiment. (B) Representative microphotographs of WT DRG dissociated cultures showing that RSK3 overexpression in naive cultures does not phenocopy the preconditioning effect. Scale bar: 250 μm. (C–E) Quantification of B. (C) Longest neurite length per neuron 16 h after plating (mean ± SEM, one-way ANOVA, at least 3 independent DRG cultures, approximately 50–100 cells counted per condition per culture). (D) Mean distance between 2 ramifications (mean ± SEM, one-way ANOVA, at least 3 independent DRG cultures, approximately 50 cells analyzed per condition per culture) and (E) percentage of neurons growing a neurite 16 h after plating (mean ± SEM, two-way ANOVA, 10 random microscopy fields quantified per condition per culture). (F) Representative confocal images of sciatic nerve sections 3 days post-injury from mice injected intrathecally with AAV8-Ctrl (control) or AAV8-RSK3. Regenerating axons are labeled with anti-SCG10 antibody (white). The red dashed line indicates the injury site. Scale bar: 500 μm. (G, H) Quantification of regenerative axons from F (mean ± SEM, multiple unpaired *t* test, at least 3 animals per group). (H) Regeneration index at 3 dpi (mean ± SEM, unpaired *t* test, at least 3 animals per group). (I) Representative microphotographs of TdTomato^fl/fl^ DRG sections stained with anti-GFP (in green) and anti-Tuj 1 (in gray) antibodies 21 days after co-intrathecal injection of AAV8-GFP (Ctrl) and AAV8-CRE. tdTomato is in magenta. Scale bar: 25 μm. (J) Quantification of I (mean ± SEM, 3 animals, 5 DRG sections counted per animal). (K) Representative confocal images of sciatic nerve sections 3 days post-injury from mice co-injected intrathecally with AAV8-Ctrl (control) and AAV8-shCtrl (control) or AAV8-CRE and AAV8-shCtrl (control). Regenerating axons are labeled with anti-SCG10 antibody (white). The red dashed line indicates the injury site. Scale bar: 500 μm. (L, M) Quantification of regenerative axons from K (mean ± SEM, multiple unpaired *t* test, at least 5 animals per group). (M) Regeneration index at 3 dpi (mean ± SEM, unpaired *t* test, at least 5 animals per group). ⁎⁎⁎*p* < 0.001, ⁎⁎*p* < 0.01, ⁎*p* < 0.05, ns: not significant. Raw data can be found in Supporting information (S1 Data and S1 Raw Images).
(TIF)

S8 Fig RSK2 is necessary for dorsal column regeneration in preconditioned condition. (A) Workflow of experiments. (B) Schematic representation of the dorsal column with representative images of cervical, thoracic, and lumbar coronal sections of mice 6 weeks after dorsal column crush at thoracic T7 level, 1 week after CTB-Alexa-555 intranervous injection in the sciatic nerve. (C) Representative confocal images of thoracic spinal cord sagittal sections 6 weeks after sciatic nerve crush and dorsal column crush from mice injected intrathecally with AAV8-sh-Scrambled or AAV8-sh-RSK2. Regenerative axons are labeled with anti-CTB antibody (white). The orange arrow shows the direction of axon regeneration. (D) Quantification of axon regeneration and dieback from caudal marge of crush site from C (mean ± SEM, Mann–Whitney test, *N* = at least 8 animals per group). (E) Von Frey experiment to test nociception in mice intrathecally injected with AAV8-shScrambled or AAV8-shRSK2, 2 weeks before and 6 weeks after sciatic nerve injury and dorsal column crush, stimulus intensity is showed in grams (mean ± SEM, multiple *t* test, at least 11–12 animals per group, only the injured paw was considered). (F, G) Tape contact and removal test in mice intrathecally injected with AAV8-shScrambled or AAV8-shRSK2, 2 weeks before and 6 weeks after left sciatic nerve injury and dorsal column crush (mean ± SEM, two-way ANOVA, at least 11 animals per group). ⁎⁎⁎*p* < 0.001, ⁎⁎*p* < 0.01, ⁎*p* < 0.05. Raw data can be found in Supporting information (S1 Data and S1 Raw Images).
(TIF)

S1 Table List of cDNAs used for in situ hybridization. (XLSX)

S2 Table List of the antibodies. (XLSX)

S1 Data The underlying data for Figs 1D, 1E, 1F, 1H, 2C, 2D, 2E, 2G, 2H, 2J, 2K, 2L, 2N, 2O, 3B, 3C, 3D, 3E, 3G, 3H, 4C, 4E, 4H, 4J, 4L, 5B, 5C, 5D, 5F, 5G, 5I, 5J, 5K, 5M, 5N, 5P, 5Q, 5R, 5T, 5U, 6B, 6C, 6D, 6F, 6G, 7D, 7F, 7G, 7H, 7J, 7L, 7N, 7Q, 7R and S1C, S1D, S2D, S1E, S2G, S2H, S2I, S3D, S3E, S3F, S3H, S3I, S3K, S3L, S3M, S4C, S4D, S6B, S6C, S6D, S7C, S7D, S7E, S7G, S7H, S7L, S7M, S8D, S8E, S8F, S8F and S8G. (XLSX)

S1 Raw Images Original blot images for Figs 1C, 4B, 4I, 4K, S2C, S3A, S3B and S6B–S6E. (PDF)

## Abbreviations

CNS: central nervous system
CTB: cholera toxin B
dpi: days post-injury
DRG: dorsal root ganglion
mTOR: mammalian target of rapamycin
PC: precontionned
PNS: peripheral nervous system
p-RPS6: phosphorylated ribosomal protein S6
RGC: retina ganglion cell
RPS6: ribosomal protein S6
vGAT: vesicular gamma aminobutyric acid transporter
Vglut1: vesicular glutamate transporter 1
