# Supplementary figures and images for: The RSK2-RPS6 axis promotes axonal regeneration in the peripheral and central nervous systems
Source: PLoS Biol. 2023 Apr 17;21(4):e3002044. doi: 10.1371/journal.pbio.3002044 (PMC10109519; doi:10.1371/journal.pbio.3002044)

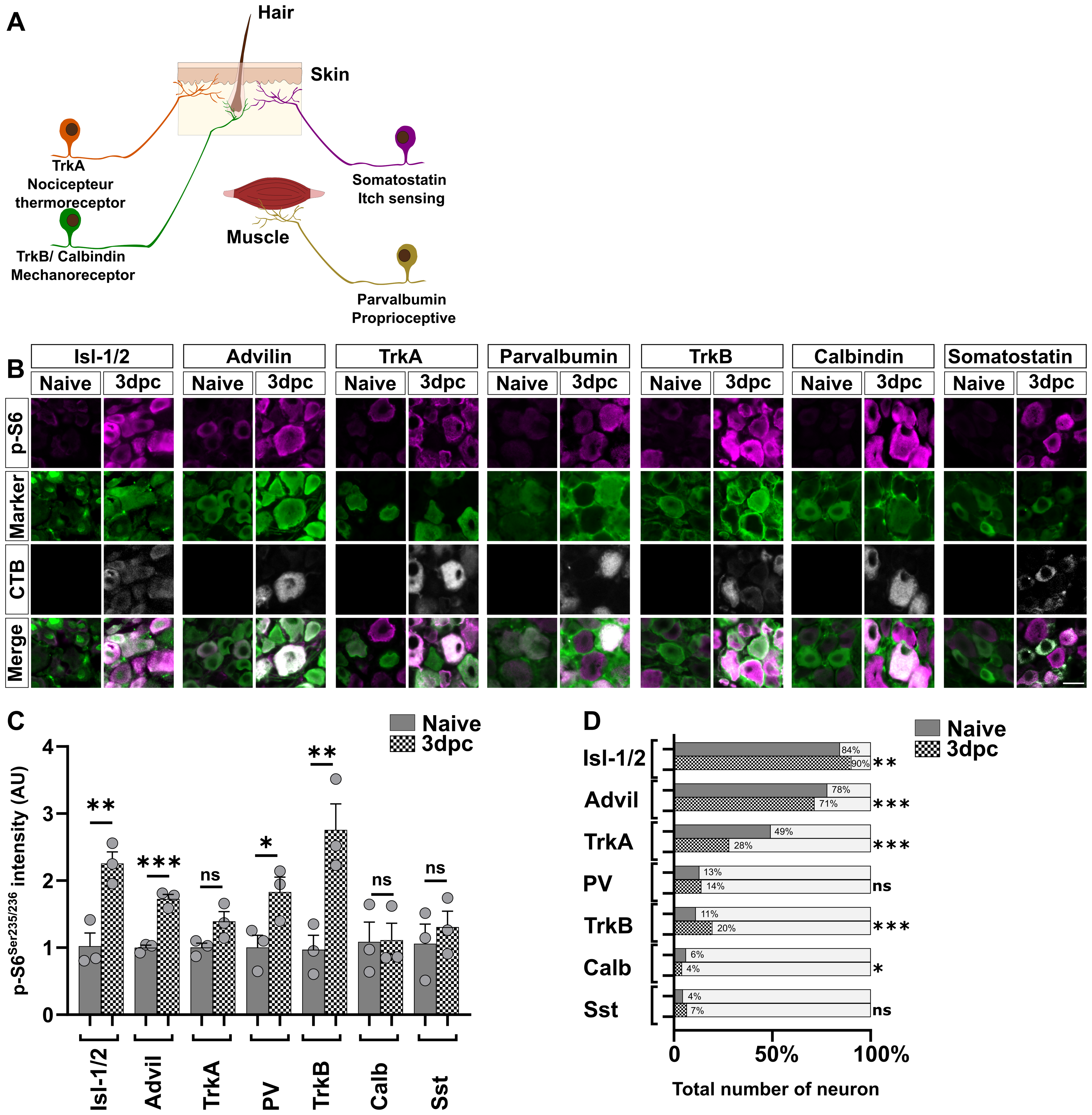

Supplement: S1 Fig — (A) Schematic illustration of sensory neuron subtypes in adult DRG, based on their functions and the markers they expressed: TrkA, TrkB, Calbindin, Somatostatin, and Parvalbumin. (B) Representative microphotographs of DRG sections stained with anti-p-S6Ser235-236 (in magenta), CTB (in gray, only at 3dpi), and different DRG subpopulations markers (in green) in intact and 3dpc. Scale bar: 50 μm. (C) Graphs showing the quantification of B with a differential up-regulation of p-S6Ser235-236 at 3-dpi among different DRG subpopulations (mean ± SEM; unpaired t test; N = 3 animals per group; at least 50 positive neurons for each marker were counted). (D) Graphs showing proportion of CTB retro-labeled subpopulations in intact DRG and their proportion 3 dpi (Chi-squared test; at least 37 positive neurons for each marker were counted). Raw data can be found in Supporting information (S1 Data). (TIF) [file pbio.3002044.s001.tif]

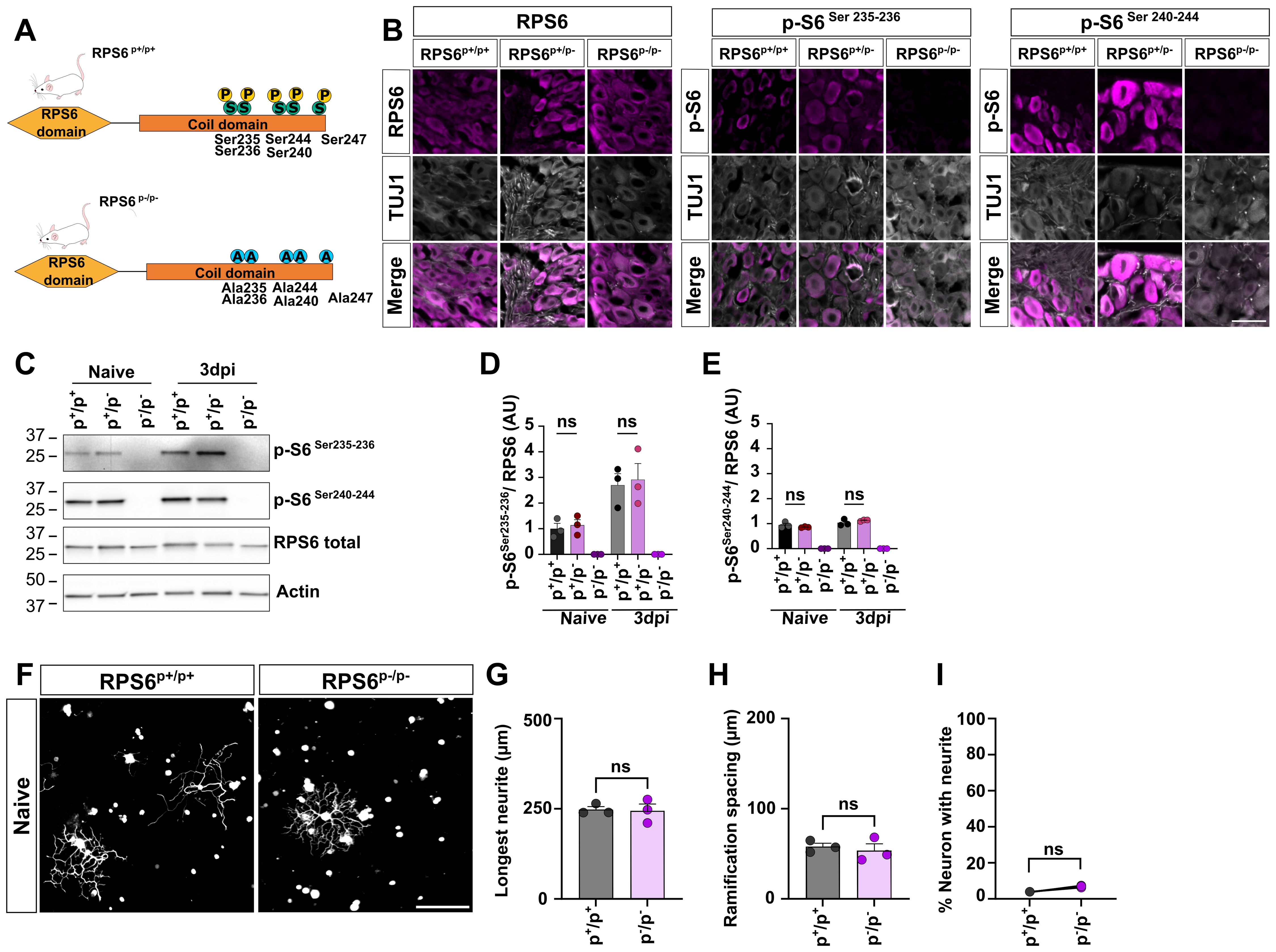

Supplement: S2 Fig — (A) Schematic describing the unphosphorylable RSP6 mouse line. (B) Representative microphotographs of DRG sections from RPS6p+/p+, RPS6p+/p-, and RPS6p-/p- stained with anti-RPS6, anti-p-S6Ser235-236, or anti-p-S6Ser240-244 (in magenta) and anti-Tuj 1 (in gray). Scale bar: 25 μm. (C) Western blot showing that RPS6 is not phosphorylated in RPS6p-/p- DRG compared to RPS6p+/p+ and RPS6p-/p+ DRG. (D, E) Quantification of C (mean ± SEM; Ordinary one-way ANOVA; N = 3 animals per group). (F) Representative microphotographs of naive cultures of mature DRG neurons from WT (RPS6p+/p+) and homozygous (RPS6p-/p-) mice line defective for RPS6 phosphorylation showing no differences. Scale bar: 250 μm. (G–I) Graphs showing the quantification of F. (G) Longest neurite length per neuron 16 h after plating (mean ± SEM, unpaired t test, 3 independent DRG cultures, approximately 50 cells counted per conditions per culture). (H) Distance between 2 ramifications in longest neurite (mean ± SEM, unpaired t test, 3 independent DRG cultures, approximately 50 cells analyzed per condition per culture). (I) Percentage of neurons growing a neurite 16 h after plating (mean ± SEM, unpaired t test, 10 random microscopy fields quantified per condition, ns: non-significant). Raw data can be found in Supporting information (S1 Data and S1 Raw Images). (TIF) [file pbio.3002044.s002.tif]

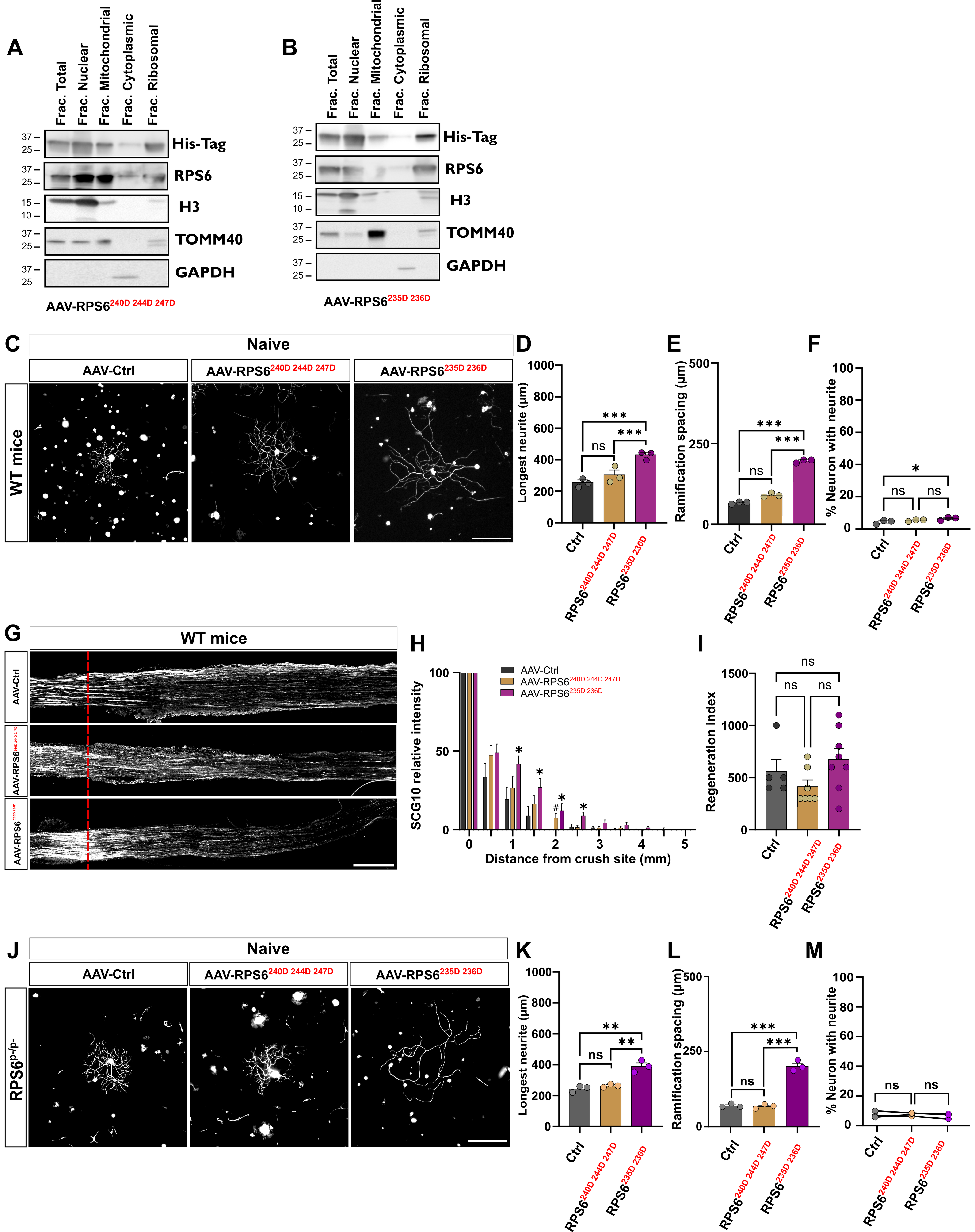

Supplement: S3 Fig — (A, B) Western blot of ribosome purification showing a good integration of phosphomimetics RPS6 constructs (A) RPS6 240D-244D-247D or (B) RPS6235D-236D in ribosome of N2A cells. (C) Representative microphotographs of naive cultures of mature DRG neurons from WT mice 21 days after intrathecal injection of AAV8-Ctrl; AAV8-RPS6240D-244D-247D or AAV8-RPS6235D-236D showing that only overexpression of AAV8-RPS6235D-236D induces the preconditioning effect. Scale bar: 250 μm. (D, E) Graphs showing the quantification of C. (D) Longest neurite length per neuron 16 h after plating (mean ± SEM, one-way ANOVA, 3 independent DRG cultures, approximately 50 cells counted per condition per culture). (E) Distance between 2 ramifications in longest neurite (mean ± SEM; one-way ANOVA, 3 independent DRG cultures, approximately 50 cells analyzed per condition per culture). (F) Percentage of neurons growing a neurite 16 h after plating (mean ± SEM, two-way ANOVA, 10 random microscopy fields were quantified per condition). (G) Representative confocal images of sciatic nerve sections 3 days post-injury from WT mice injected intrathecally with AAV8-PLAP (control), AAV8-RPS6240D-244D-247D, or AAV8-RPS6235D-236D. Regenerating axons are labeled with anti-SCG10 antibody (white). The red dashed line indicates the injury site. Scale bar: 500 μm. (H) Quantification of regenerative axons from G (mean ± SEM, two-way ANOVA, at least 5 animals per group). (I) Regeneration index at 3 dpi (mean ± SEM, one-way ANOVA, at least 5 animals per group). (J) Representative microphotographs of mature naive DRG neurons cultures from RPS6p-/p- mice, 21 days after intrathecal injection of AAV8-Ctrl; AAV8- RPS6240D-244D-247D or AAV8-RPS6235D-236D showing that only overexpression of phosphomimic AAV8-RPS6235D-236D induces the preconditioning effect. Scale bar: 250 μm. (K–M) Graphs showing the quantification of J. (K) Longest neurite length per neuron 16 h after plating (mean ± SEM, one-way ANOVA, 3 independent DRG cul [file pbio.3002044.s003.tif]

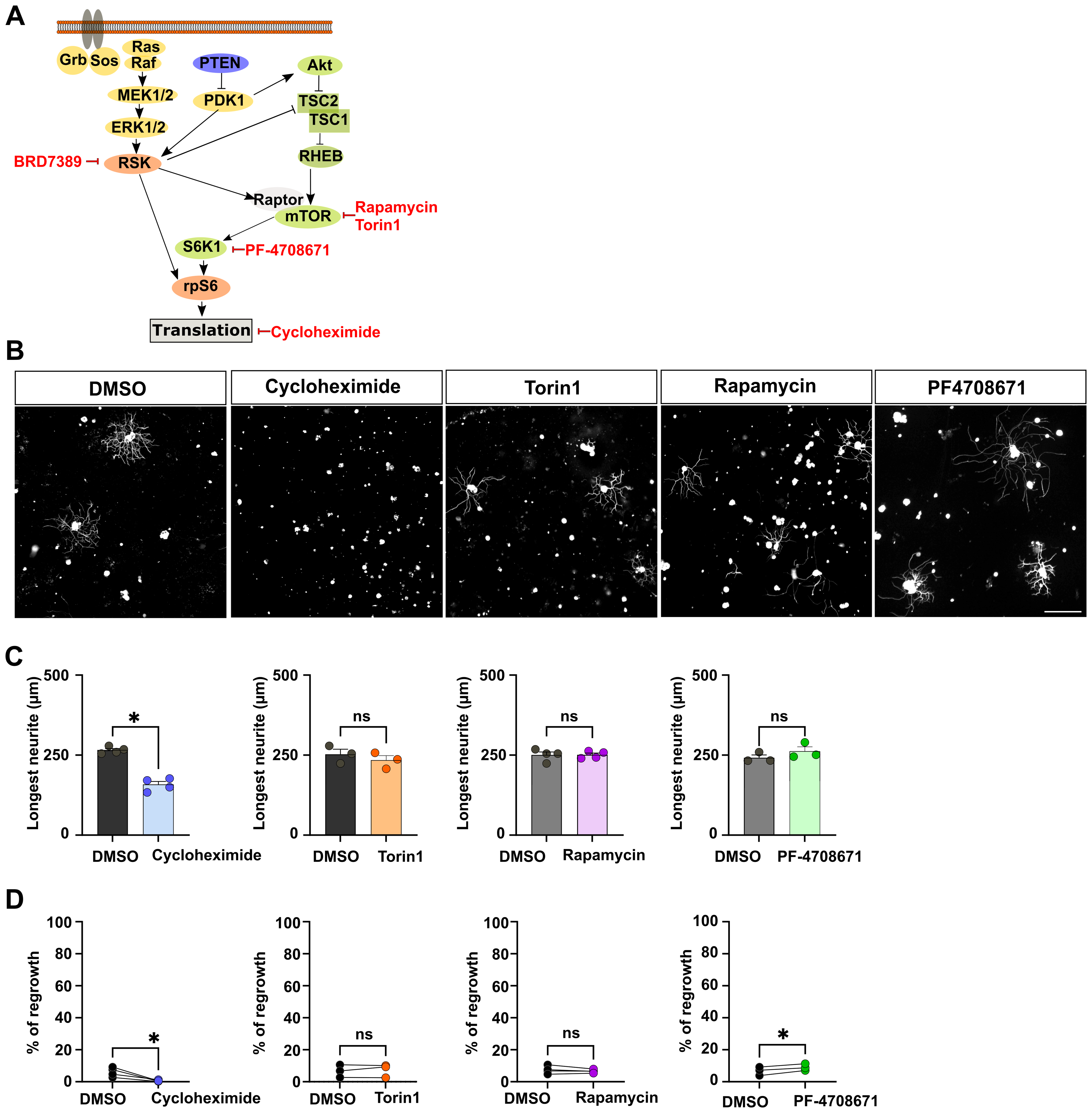

Supplement: S4 Fig — (A) Ribosomal S6 kinase schematic signaling pathway and inhibitors (in red) used in this study. (B) Representative microphotographs of naive DRG neurons cultures treated with DMSO (control), a global protein translation inhibitor (cycloheximide (5 nM)); mTOR inhibitors (Torin1 (5 nM) or Rapamycin (0.1 nM)); and an S6K1 inhibitor (PF-4708671 (8 μm)). Scale bar: 250 μm. (C) Quantification of B (mean ± SEM, two-way ANOVA, 3–4 independent DRG cultures, approximately 50–100 cells counted per condition per culture (except for cycloheximide)). (D) Percentage of neurons growing a neurite 16 h after plating from A (mean, two-way ANOVA, 3–4 independent DRG cultures, 10 random microscopy fields were quantified per condition). ⁎⁎⁎p < 0.001, ⁎⁎p < 0.01, ⁎p < 0.05, ns: not significant. Raw data can be found in Supporting information (S1 Data). (TIF) [file pbio.3002044.s004.tif]

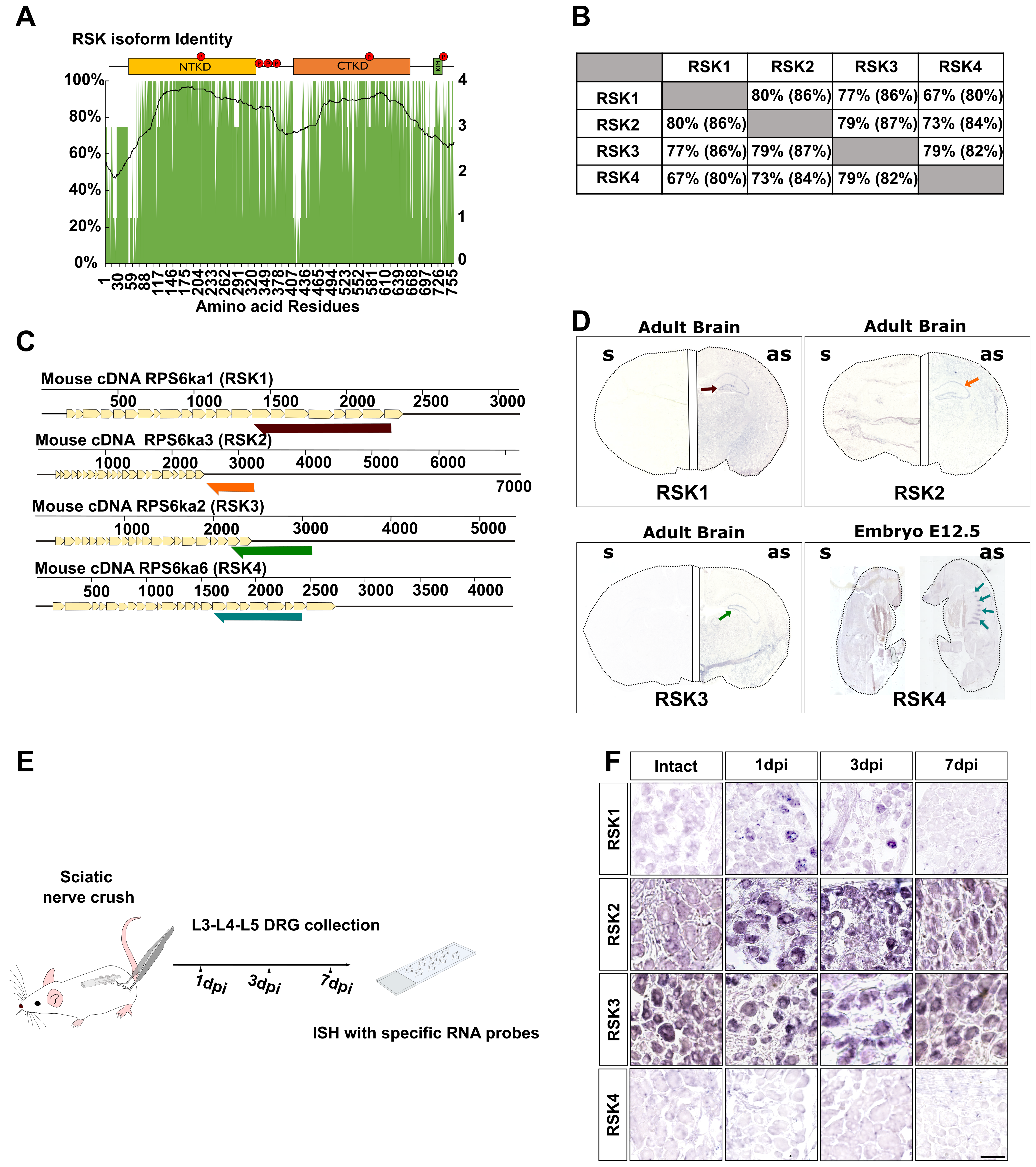

Supplement: S5 Fig — (A) Graph showing the homology of amino acid sequences among the 4 RSK expressed in mouse. (B) Table summarizing the homology and identity among RSK1, 2, 3, and 4. (C) Schematic of the probes used to study specific expression of RSK1, 2, 3, and 4 by in situ hybridization. (D) Microphotographs showing in situ hybridization with sense and anti-sense RNA probes of RSK1, RSK2, RSK3 on adult brain coronal sections and RSK4 on embryonic E12.5 sagittal section showing specificity of these probes. (E) Workflow of experiment. (F) Microphotographs showing in situ hybridization of RSK1, RSK2, RSK3, and RSK4 on adult lumbar DRG sections in intact and after sciatic injury at 1, 3, and 7 days post-injury (dpi). Only RSK2 and RSK3 are highly expressed in mouse lumbar DRG and RSK2 expression is regulated by axon injury. Scale bar: 50 μm. (TIF) [file pbio.3002044.s005.tif]

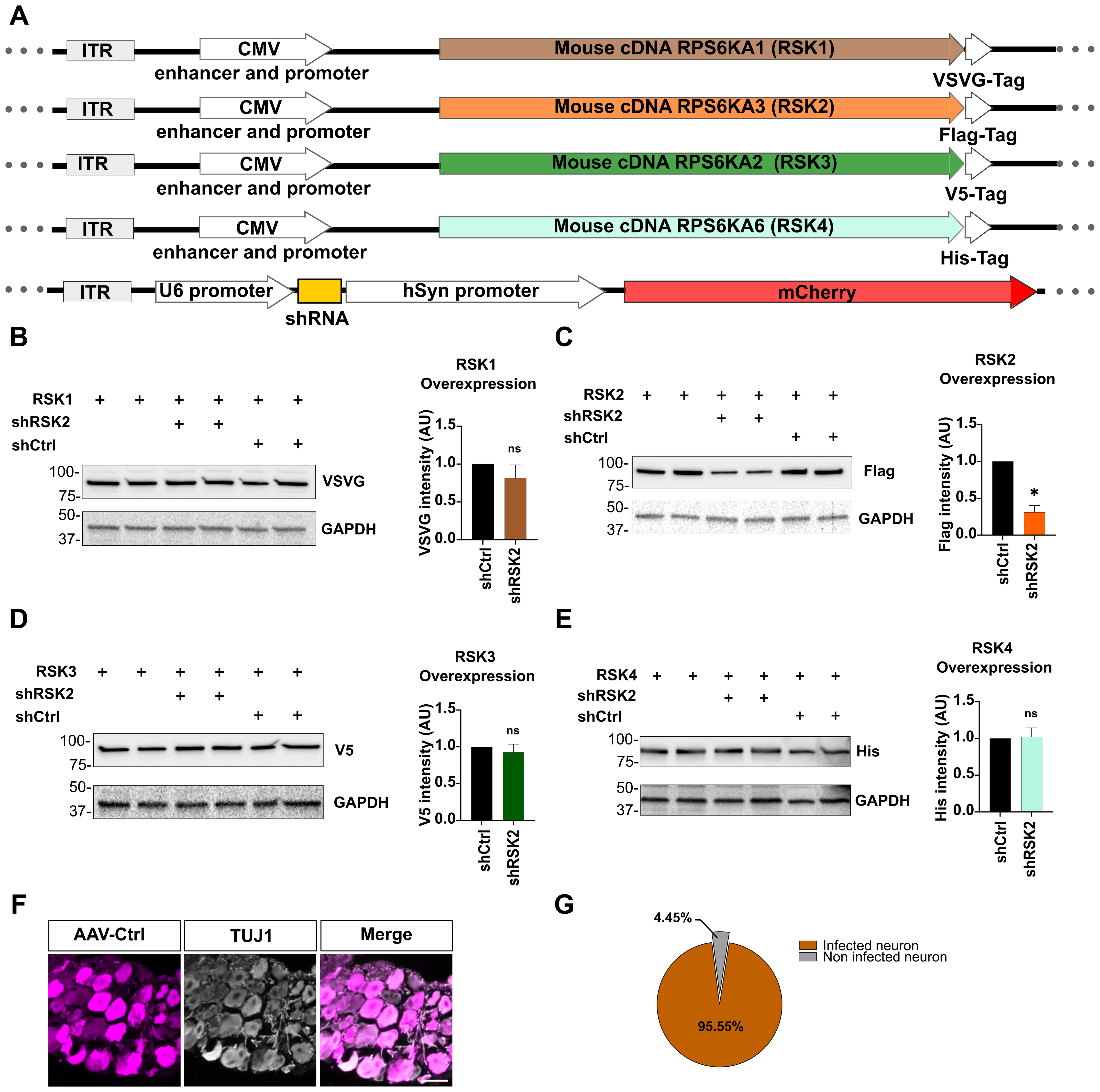

Supplement: S6 Fig — (A) Schematic of the plasmid constructs used to overexpress RSK1-VSVG, RSK2-Flag, RSK3-V5, RSK4-His, PLAP, or shRNA (sh-Scrambled or sh-RSK2). (B–E) Western blot showing shRNA-RSK2 specificity in N2A cells 96 h after co-transfection (mean ± SEM, one-sample t test, N = 3 transfections per group). (F) Representative microphotographs of DRG sections stained with anti-RFP (in magenta) and anti-Tuj 1 (in gray) antibodies, 21 days after intrathecal injection of AAV8-shCtrl (that co expressed the RFP). Scale bar: 25 μm. (G) Quantification of H (mean ± SEM, 3 animals, 5 DRG sections counted per animal). ⁎⁎⁎p < 0.001, ⁎⁎p < 0.01, ⁎p < 0.05. Raw data can be found in Supporting information (S1 Data and S1 Raw Images). (TIF) [file pbio.3002044.s006.tif]

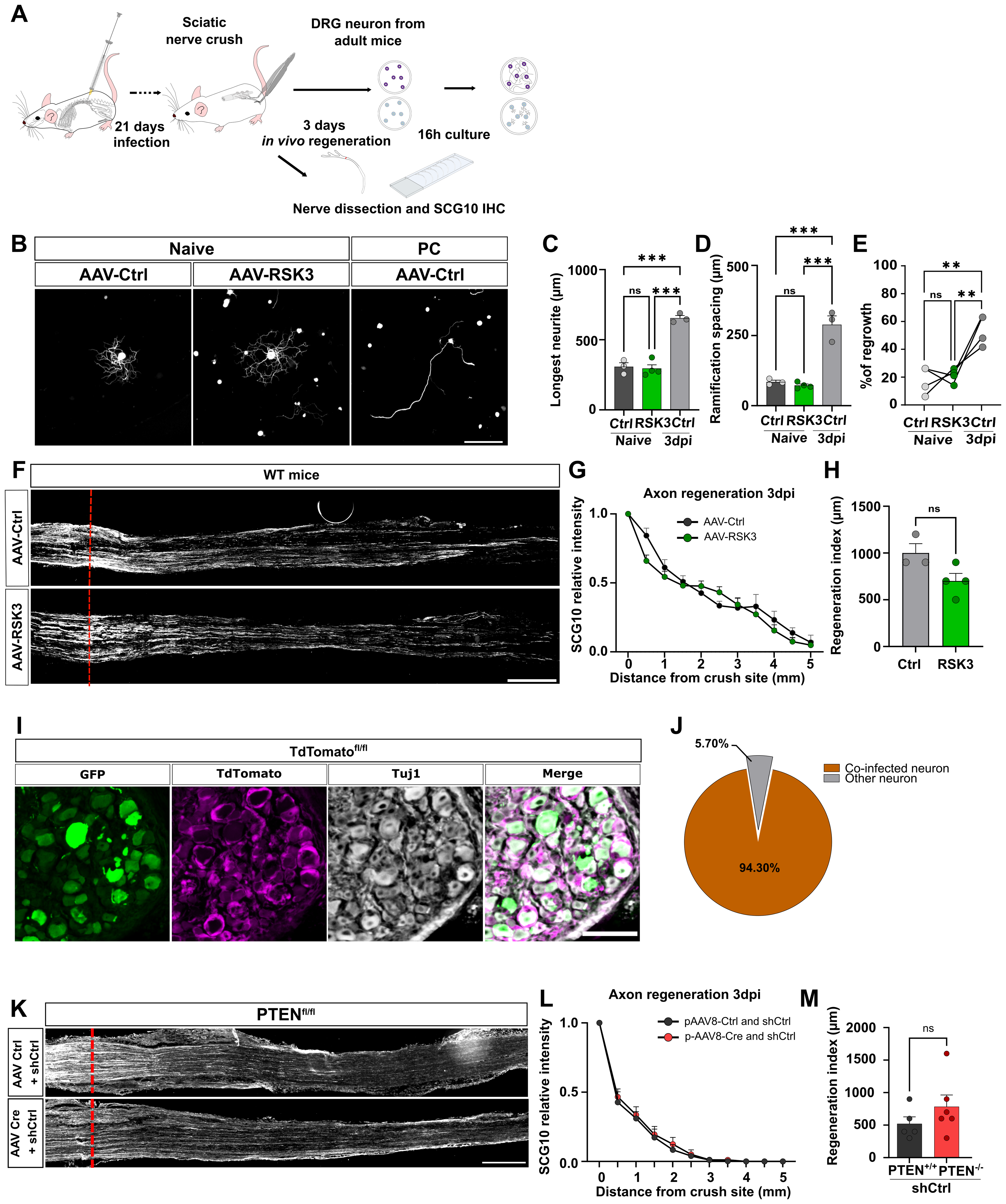

Supplement: S7 Fig — (A) Workflow of experiment. (B) Representative microphotographs of WT DRG dissociated cultures showing that RSK3 overexpression in naive cultures does not phenocopy the preconditioning effect. Scale bar: 250 μm. (C–E) Quantification of B. (C) Longest neurite length per neuron 16 h after plating (mean ± SEM, one-way ANOVA, at least 3 independent DRG cultures, approximately 50–100 cells counted per condition per culture). (D) Mean distance between 2 ramifications (mean ± SEM, one-way ANOVA, at least 3 independent DRG cultures, approximately 50 cells analyzed per condition per culture) and (E) percentage of neurons growing a neurite 16 h after plating (mean ± SEM, two-way ANOVA, 10 random microscopy fields quantified per condition per culture). (F) Representative confocal images of sciatic nerve sections 3 days post-injury from mice injected intrathecally with AAV8-Ctrl (control) or AAV8-RSK3. Regenerating axons are labeled with anti-SCG10 antibody (white). The red dashed line indicates the injury site. Scale bar: 500 μm. (G, H) Quantification of regenerative axons from F (mean ± SEM, multiple unpaired t test, at least 3 animals per group). (H) Regeneration index at 3 dpi (mean ± SEM, unpaired t test, at least 3 animals per group). (I) Representative microphotographs of TdTomatofl/fl DRG sections stained with anti-GFP (in green) and anti-Tuj 1 (in gray) antibodies 21 days after co-intrathecal injection of AAV8-GFP (Ctrl) and AAV8-CRE. tdTomato is in magenta. Scale bar: 25 μm. (J) Quantification of I (mean ± SEM, 3 animals, 5 DRG sections counted per animal). (K) Representative confocal images of sciatic nerve sections 3 days post-injury from mice co-injected intrathecally with AAV8-Ctrl (control) and AAV8-shCtrl (control) or AAV8-CRE and AAV8-shCtrl (control). Regenerating axons are labeled with anti-SCG10 antibody (white). The red dashed line indicates the injury site. Scale bar: 500 μm. (L, M) Quantification of regenerative axons from K (mean ± SEM, multiple unpaired [file pbio.3002044.s007.tif]

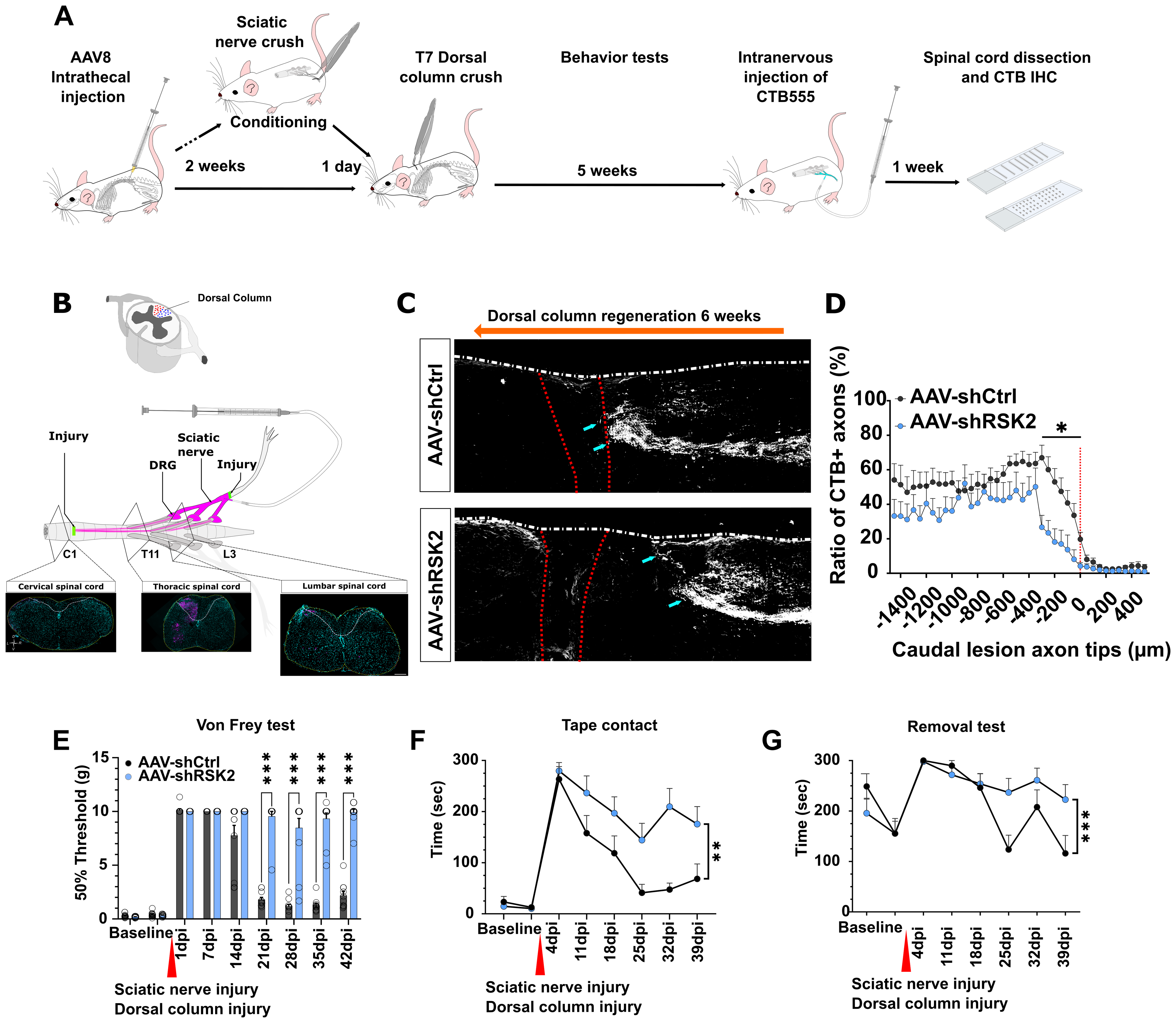

Supplement: S8 Fig — (A) Workflow of experiments. (B) Schematic representation of the dorsal column with representative images of cervical, thoracic, and lumbar coronal sections of mice 6 weeks after dorsal column crush at thoracic T7 level, 1 week after CTB-Alexa-555 intranervous injection in the sciatic nerve. (C) Representative confocal images of thoracic spinal cord sagittal sections 6 weeks after sciatic nerve crush and dorsal column crush from mice injected intrathecally with AAV8-sh-Scrambled or AAV8-sh-RSK2. Regenerative axons are labeled with anti-CTB antibody (white). The orange arrow shows the direction of axon regeneration. (D) Quantification of axon regeneration and dieback from caudal marge of crush site from C (mean ± SEM, Mann–Whitney test, N = at least 8 animals per group). (E) Von Frey experiment to test nociception in mice intrathecally injected with AAV8-shScrambled or AAV8-shRSK2, 2 weeks before and 6 weeks after sciatic nerve injury and dorsal column crush, stimulus intensity is showed in grams (mean ± SEM, multiple t test, at least 11–12 animals per group, only the injured paw was considered). (F, G) Tape contact and removal test in mice intrathecally injected with AAV8-shScrambled or AAV8-shRSK2, 2 weeks before and 6 weeks after left sciatic nerve injury and dorsal column crush (mean ± SEM, two-way ANOVA, at least 11 animals per group). ⁎⁎⁎p < 0.001, ⁎⁎p < 0.01, ⁎p < 0.05. Raw data can be found in Supporting information (S1 Data and S1 Raw Images). (TIF) [file pbio.3002044.s008.tif]
