# Supplementary material for: The RSK2-RPS6 axis promotes axonal regeneration in the peripheral and central nervous systems
Source: PLoS Biol. 2023 Apr 17;21(4):e3002044. doi: 10.1371/journal.pbio.3002044 (PMC10109519; doi:10.1371/journal.pbio.3002044)
Supplement: S1 Raw Images — (PDF) [file pbio.3002044.s012.pdf]

S1_raw_images. Original blot images for 1C, 4B, 4L, 4K, S2C, S3A, S3B, S6B-E

Fig 1C

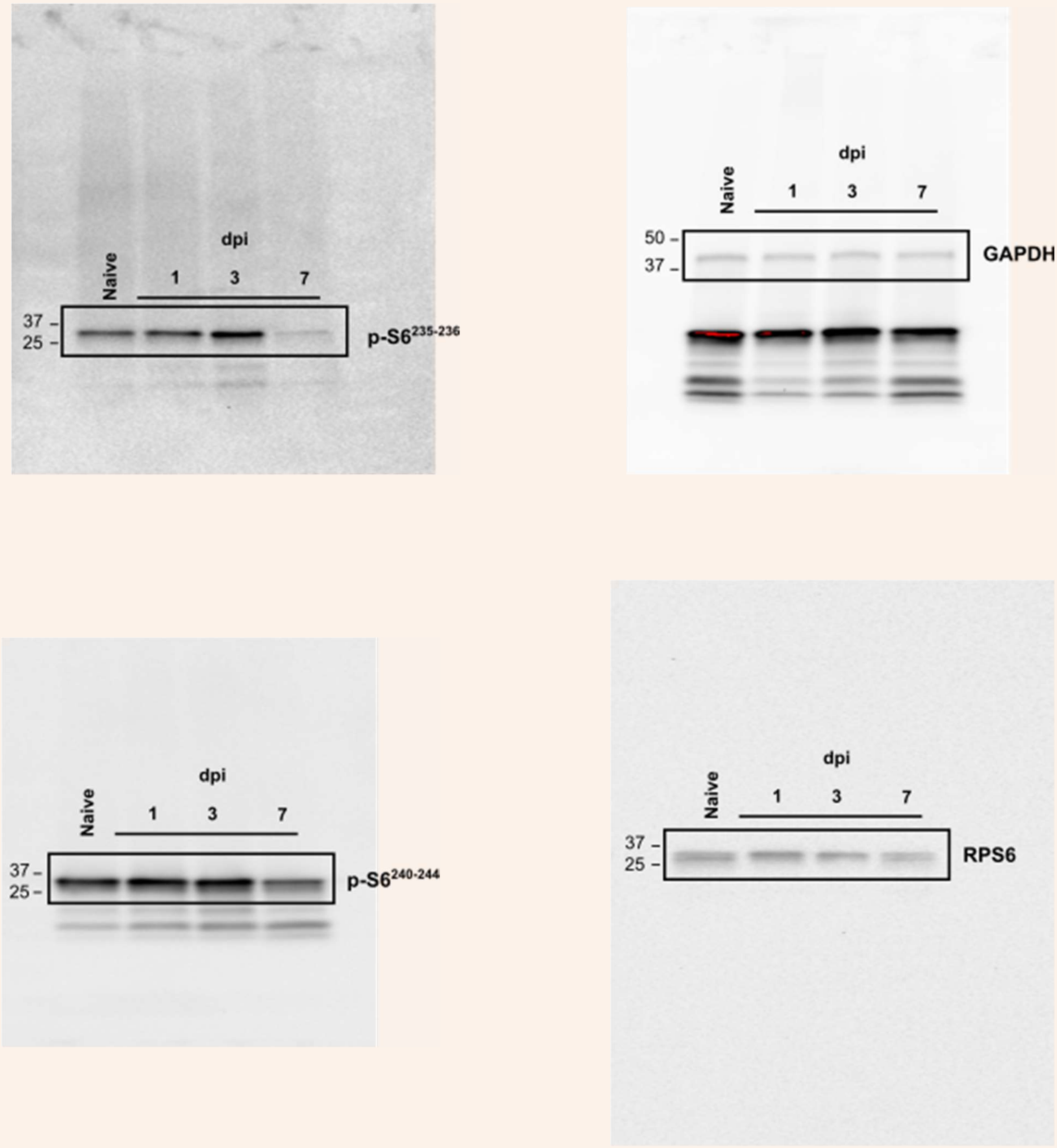
dpi
Naive
1
3
7
37
25
p-S6235-236
dpi
Naive
1
3
7
50
37
GAPDH
dpi
Naive
1
3
7
37
25
p-S6240-244
dpi
Naive
1
3
7
37
25
RPS6

fig 4B

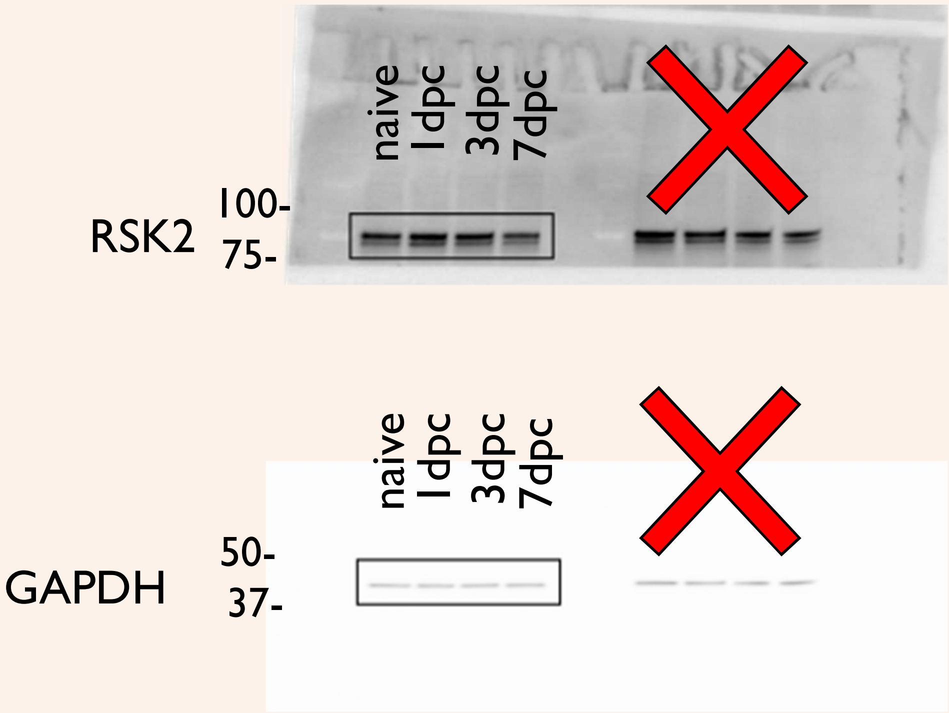

naive
1dpc
3dpc
7dpc
RSK2
100-
75-
naive
1dpc
3dpc
7dpc
GAPDH
50-
37-

fig 4I

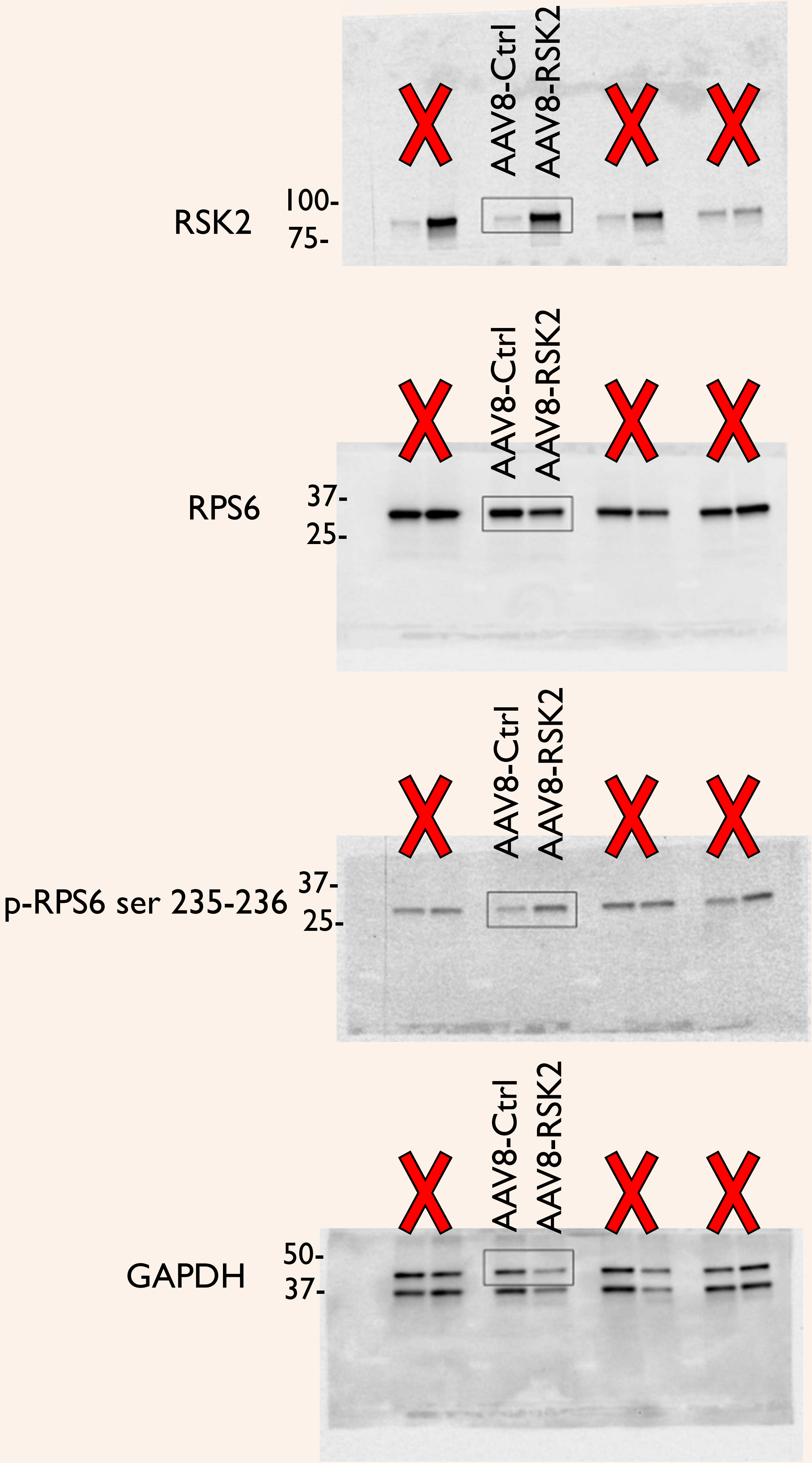

AAV8-Ctrl
AAV8-RSK2
RSK2
100-
75-
AAV8-Ctrl
AAV8-RSK2
RPS6
37-
25-
AAV8-Ctrl
AAV8-RSK2
p-RPS6 ser 235-236
37-
25-
AAV8-Ctrl
AAV8-RSK2
GAPDH
50-
37-

fig 4K

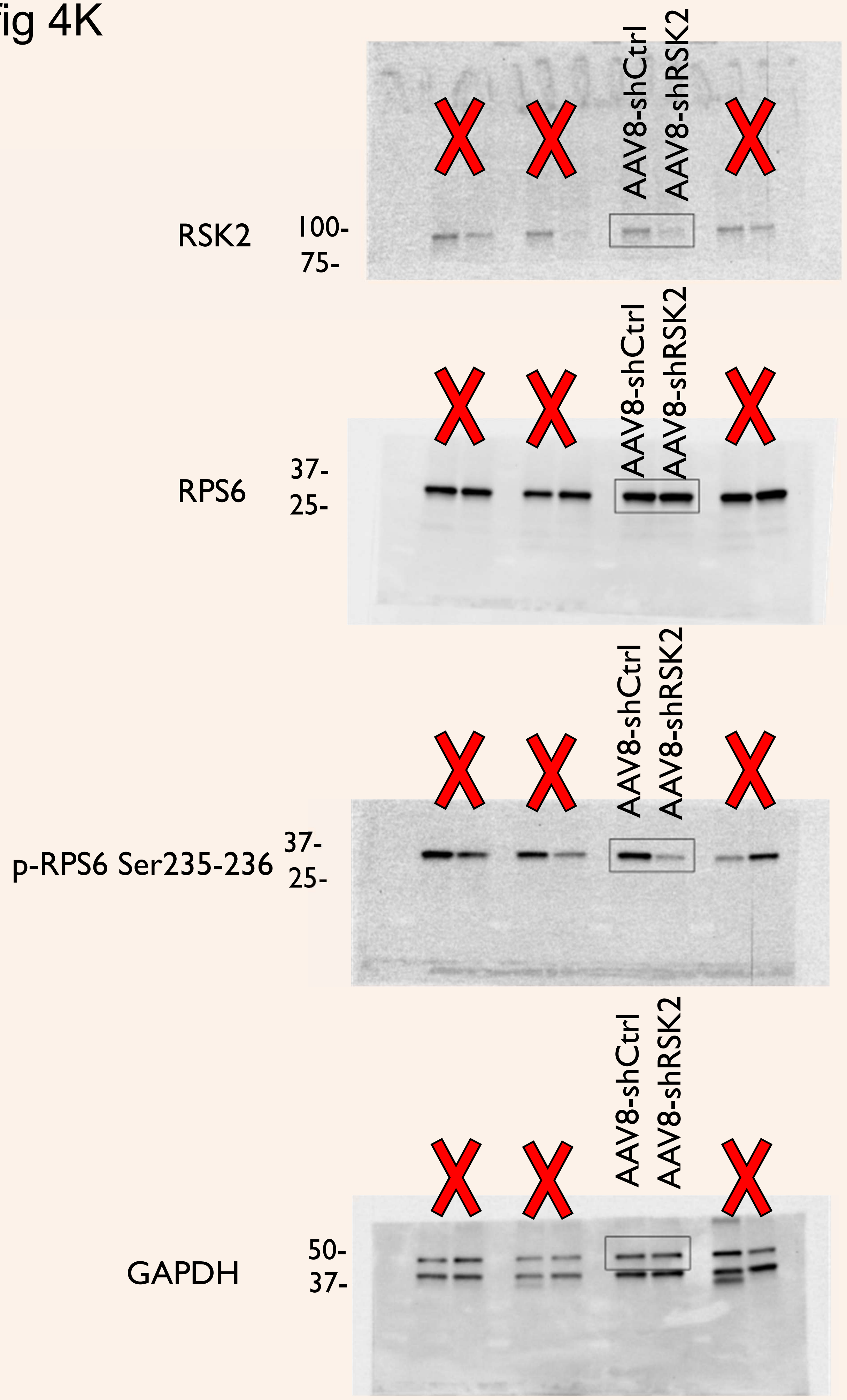
AAV8-shCtrl
AAV8-shRSK2
RSK2
100-
75-
RPS6
37-
25-
p-RPS6 Ser235-236
37-
25-
GAPDH
50-
37-

fig S2C

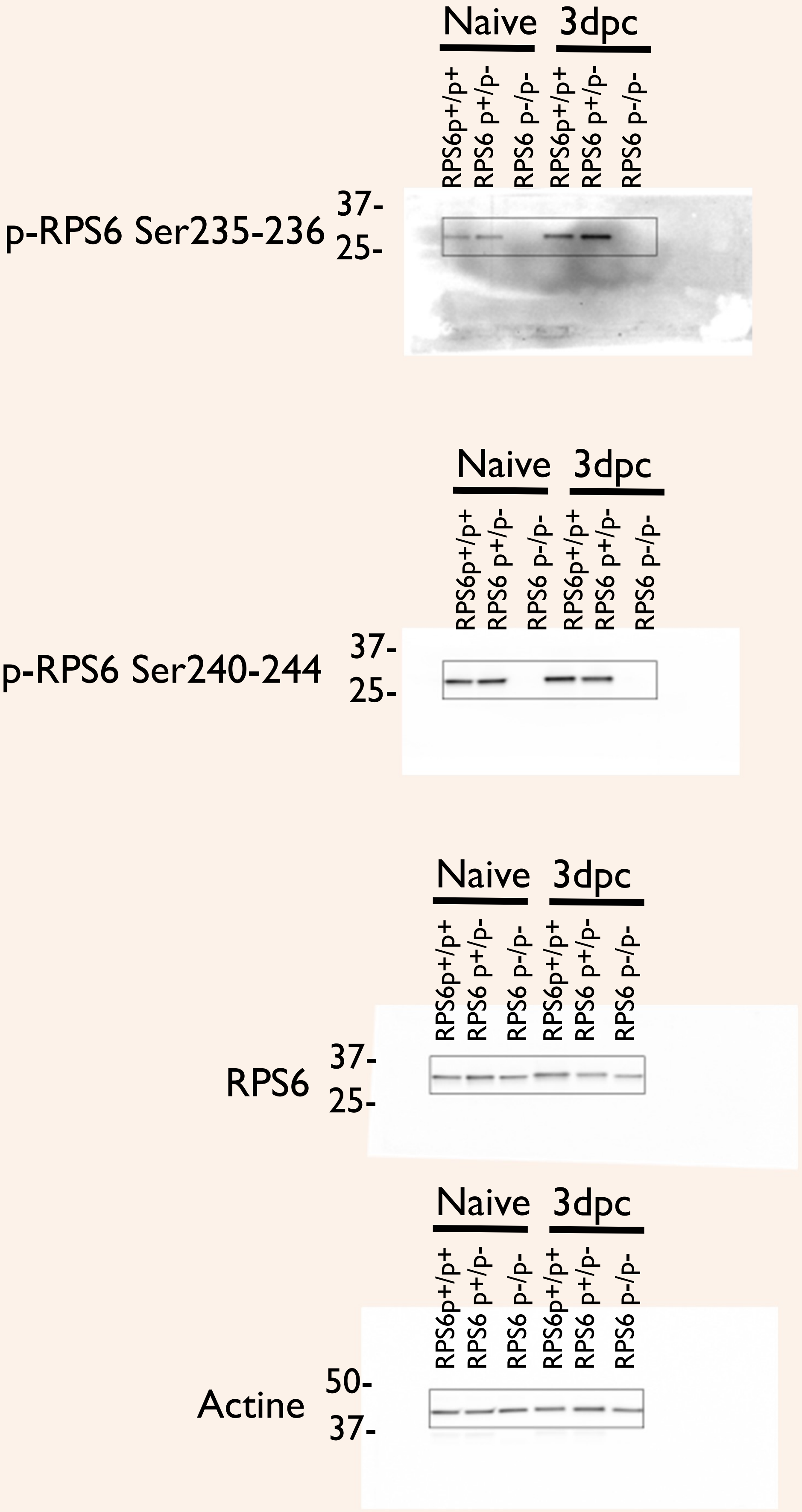
Naive
3dpc
RPS6p+/p+
RPS6 p+/p-
RPS6 p-/p-
RPS6p+/p+
RPS6 p+/p-
RPS6 p-/p-
p-RPS6 Ser235-236
37-
25-
Naive
3dpc
RPS6p+/p+
RPS6 p+/p-
RPS6 p-/p-
RPS6p+/p+
RPS6 p+/p-
RPS6 p-/p-
p-RPS6 Ser240-244
37-
25-
Naive
3dpc
RPS6p+/p+
RPS6 p+/p-
RPS6 p-/p-
RPS6p+/p+
RPS6 p+/p-
RPS6 p-/p-
RPS6
37-
25-
Naive
3dpc
RPS6p+/p+
RPS6 p+/p-
RPS6 p-/p-
RPS6p+/p+
RPS6 p+/p-
RPS6 p-/p-
Actine
50-
37-

fig S3A

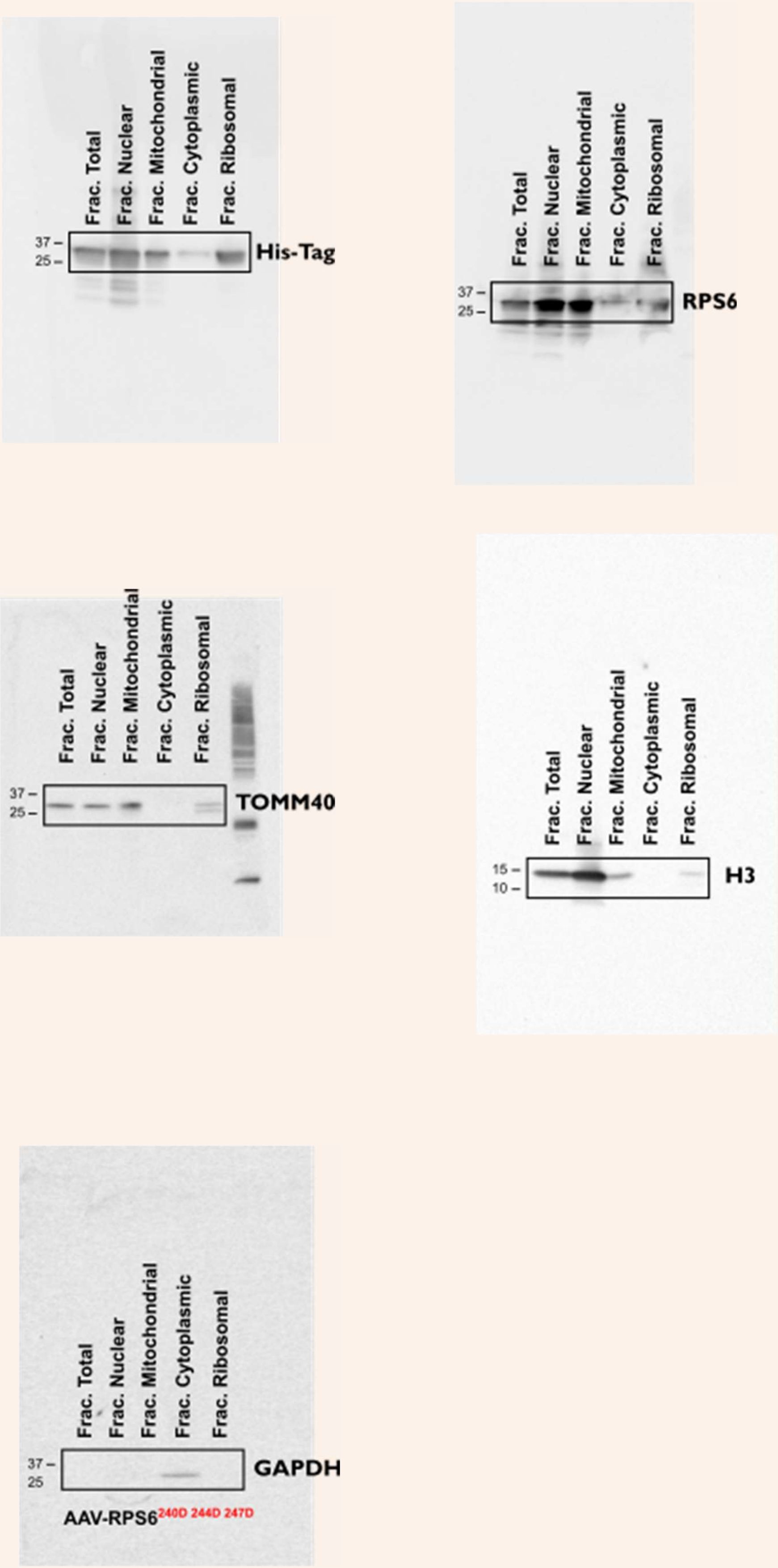
Frac. Total
Frac. Nuclear
Frac. Mitochondrial
Frac. Cytoplasmic
Frac. Ribosomal
37 –
25 –
His-Tag
Frac. Total
Frac. Nuclear
Frac. Mitochondrial
Frac. Cytoplasmic
Frac. Ribosomal
37 –
25 –
RPS6
Frac. Total
Frac. Nuclear
Frac. Mitochondrial
Frac. Cytoplasmic
Frac. Ribosomal
37 –
25 –
TOMM40
Frac. Total
Frac. Nuclear
Frac. Mitochondrial
Frac. Cytoplasmic
Frac. Ribosomal
15 –
10 –
H3
Frac. Total
Frac. Nuclear
Frac. Mitochondrial
Frac. Cytoplasmic
Frac. Ribosomal
37 –
25
GAPDH
AAV-RPS6 240D 244D 247D

fig S3B

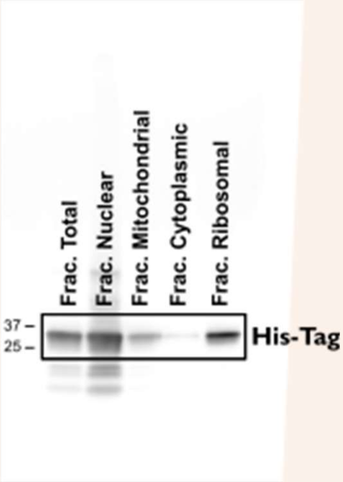

Frac. Total
Frac. Nuclear
Frac. Mitochondrial
Frac. Cytoplasmic
Frac. Ribosomal
37
25
His-Tag

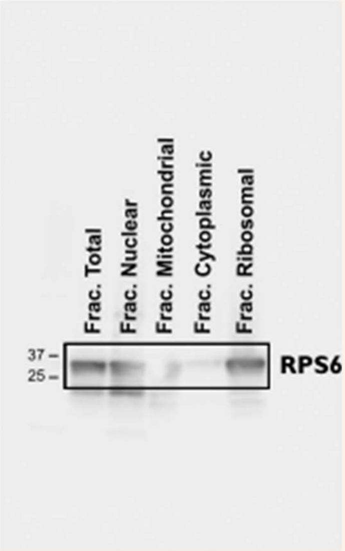

Frac. Total
Frac. Nuclear
Frac. Mitochondrial
Frac. Cytoplasmic
Frac. Ribosomal
37
25
RPS6

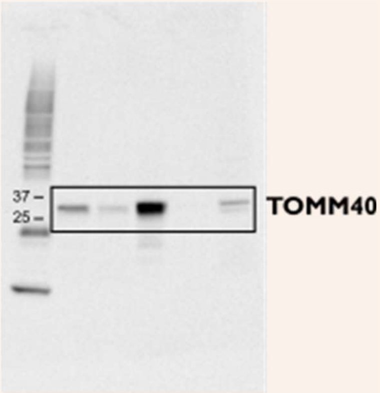

37
25
TOMM40

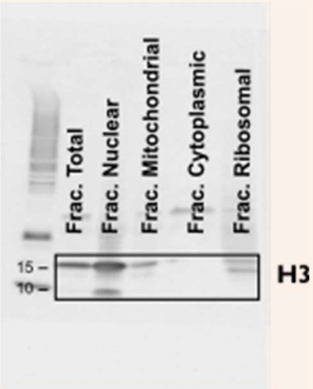

Frac. Total
Frac. Nuclear
Frac. Mitochondrial
Frac. Cytoplasmic
Frac. Ribosomal
15
10
H3

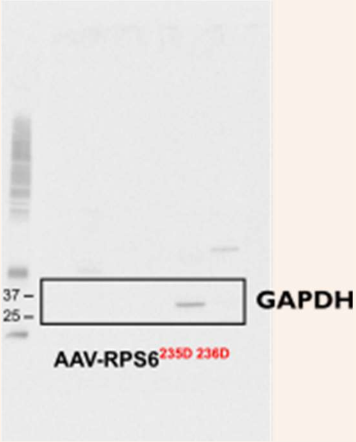

37
25
GAPDH
AAV-RPS6 235D 236D

fig S6B-E

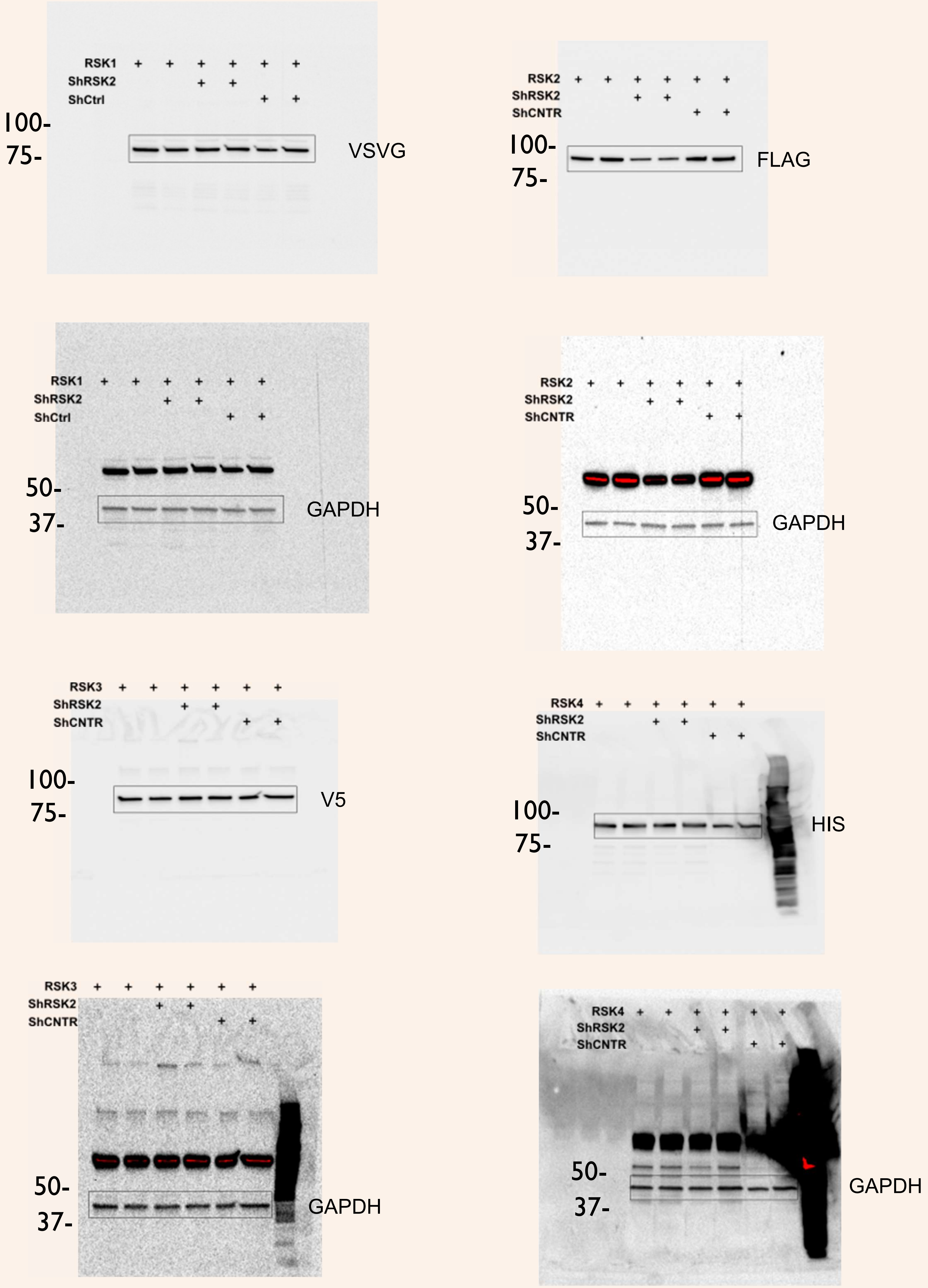
RSK1 + + + + + +
ShRSK2 + +
ShCtrl + +
100-
75-
VSVG
RSK2 + + + + + +
ShRSK2 + +
ShCNTR + +
100-
75-
FLAG
RSK1 + + + + + +
ShRSK2 + +
ShCtrl + +
50-
37-
GAPDH
RSK2 + + + + + +
ShRSK2 + +
ShCNTR + +
50-
37-
GAPDH
RSK3 + + + + + +
ShRSK2 + +
ShCNTR + +
100-
75-
V5
RSK4 + + + + + +
ShRSK2 + +
ShCNTR + +
100-
75-
HIS
RSK3 + + + + + +
ShRSK2 + +
ShCNTR + +
50-
37-
GAPDH
RSK4 + + + + + +
ShRSK2 + +
ShCNTR + +
50-
37-
GAPDH
